# Supplementary material for: Cusp-singularity-enhanced Coriolis effect for sensitive chip-scale gyroscopes
Source: Nature. 2026 May 20;653(8115):700–6. doi: 10.1038/s41586-026-10565-w (PMC13190237; doi:10.1038/s41586-026-10565-w)
Supplement: Supplementary file 1 — Supplementary Information [file 41586_2026_10565_MOESM1_ESM.pdf]

nature portfolio

https://doi.org/10.1038/s41586-026-10565-w

Supplementary information

Cusp-singularity-enhanced Coriolis effect for sensitive chip-scale gyroscopes

In the format provided by the authors and unedited

Cusp-singularity-enhanced Coriolis effect for sensitive chip-scale gyroscopes

Sen Zhang[1], Dingbang Xiao[1], Fei Wang[2*], Ran Huang[3], Lei Yu[4], Ning Zhou[4], Kaixuan He[4], Xuezhong Wu[1], Franco Nori[3*], Hui Jing[5,6*] & Xin Zhou[1,2*]

[1]College of Intelligence Science and Technology, NUDT, Changsha, 410073, China.

[2]School of Microelectronics, Southern University of Science and Technology (SUSTech), Shenzhen, 518055, China.

[3]Center for Quantum Computing (RQC), RIKEN, Wakoshi, Saitama, 351-0198, Japan.

[4]East China Institute of Photo-Electronic IC, Bengbu, 233042, China.

[5]Institute for Quantum Science and Technology, College of Science, NUDT, Changsha, 410073, China.

[6]Key Laboratory of Low-Dimensional Quantum Structures and Quantum Control of Ministry of Education, Hunan Normal University, Changsha, 410081, China.

*e-mail: wangf@sustech.edu.cn; fnori@riken.jp; jinghui73@foxmail.com; zhouxin11@nudt.edu.cn.

CONTENTS

SUPPLEMENTARY NOTE 1. Coriolis effect 3

A. General introduction 3

B. Ideal Coriolis interaction 3

C. Intrinsic Coriolis factor 4

D. Hamiltonian of the Coriolis coupled system 7

SUPPLEMENTARY NOTE 2. Stiffness coupling introduced by off-axis tuning 9

SUPPLEMENTARY NOTE 3. Dynamics of the coupled gyroscope with quadrature drive 10

A. Equations of motion 10

B. Steady-states from the quadrature drive 12

C. Phase-tracked closed-loop oscillation 13

SUPPLEMENTARY NOTE 4. Stability analysis 15

SUPPLEMENTARY NOTE 5. Cusp catastrophe 17

SUPPLEMENTARY NOTE 6. State information 18

SUPPLEMENTARY NOTE 7. Description in traveling-wave basis 20

SUPPLEMENTARY NOTE 8. Boosted frequency modulation near cusp-singularities 22

SUPPLEMENTARY NOTE 9. Cusp-singularity-mediated phase modulation 23

SUPPLEMENTARY NOTE 10. Angular-velocity estimation 26

SUPPLEMENTARY NOTE 11. Brownian noise model for singularity-enhanced frequency and phase modulations 27

SUPPLEMENTARY NOTE 12. Performance limit of amplitude-modulated operation 29

A. Angle-random-walk limit of amplitude-modulated operation 30

B. Bias-stability limit of AM operation 30

SUPPLEMENTARY REFERENCES. 31

SUPPLEMENTARY NOTE 1. CORIOLIS EFFECT

A. General introduction

The Coriolis effect refers to the perceived deviation of a moving object within a rotating reference frame. This deviation in the object's path is caused by a pseudo force known as the Coriolis force, which acts perpendicular to the object's direction of motion. The Coriolis force can be represented mathematically as

$$F_{\text{Coriolis}} = -2m\boldsymbol{\Omega} \times \boldsymbol{v},$$

where m denotes the object's mass, $\boldsymbol{v}$ its velocity, and $\boldsymbol{\Omega}$ the angular velocity of the rotating frame.

Grasping the Coriolis effect is important, for instance, it allows us to explain various complex weather phenomena in meteorology. Moreover, it has been engineered as a rotational inertial sensor, known as a Coriolis vibratory gyroscope (CVG), which is extensively utilized for detecting rotations in billions of devices, ranging from smartphones and automobiles [1, 2] to the James Webb Space Telescope [3]. In what follows, we will introduce the Coriolis effect-induced interaction in the CVG and the geometric confinement imposed by the Coriolis factor.

B. Ideal Coriolis interaction

We first examine an ideal gyroscopic model depicted by a proof mass supported by two orthogonal sets of springs in Fig. 1a of the main text (excluding the green off-axis spring). We focus on an out-of-plane rotation with angular velocity $\boldsymbol{\Omega} = (0, 0, \Omega)^{\text{T}}$. In the device reference frame, mode 1 (2) induces a Coriolis force of $-2m\Omega\dot{q}_1$ ($2m\Omega\dot{q}_2$) on mode 2 (1). The Newtonian motion equations are expressed as

$$\begin{aligned} m\ddot{q}_1 + m\gamma\dot{q}_1 + m\omega_1^2 q_1 - 2m\Omega\dot{q}_2 = 0, \\ m\ddot{q}_2 + m\gamma\dot{q}_2 + m\omega_2^2 q_2 + 2m\Omega\dot{q}_1 = 0, \end{aligned} \tag{S.1}$$

where $\omega_{1,2}$ represent the natural frequencies and γ denotes the damping rate of the modes.

In this context, the Coriolis mass (denoted as m in $\pm 2m\Omega\dot{q}_{1,2}$) is defined differently from the inertial mass (the m in $m\ddot{q}_{1,2}$). They are equal only in the ideal lumped-mass model, where the proof mass is rigid and springs are massless. However, it will be demonstrated that typically, the Coriolis mass is less than the inertial mass, resulting in a reduction of the Coriolis force, quantified by the intrinsic Coriolis factor $\kappa_0 \leq 1$.

C. Intrinsic Coriolis factor

This section provides a comprehensive description of the origin of the intrinsic Coriolis factor κ_0, highlighting its connection to the geometry of deformation in associated vibrational modes.

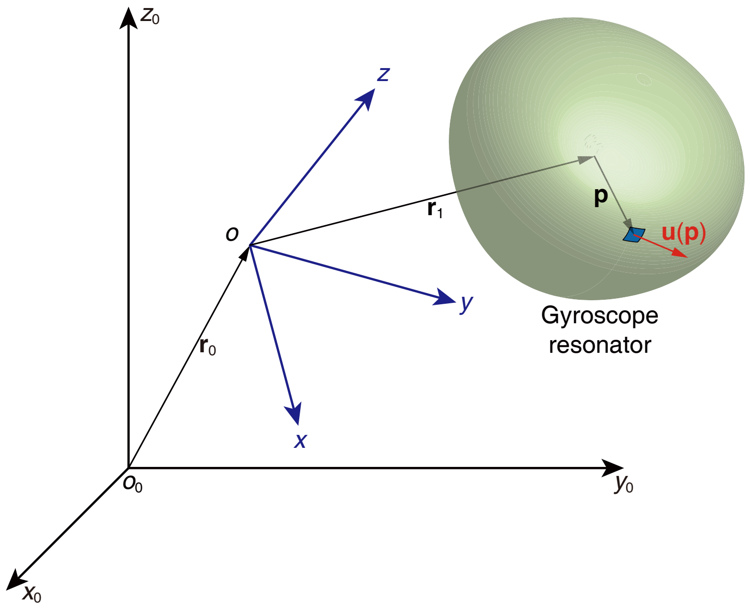

Supplementary Figure 1. **Generalized gyroscope model with specified coordinates.** The coordinates x_0-y_0-z_0 denote the inertial frame of reference. The gyroscope resonator is fixed to a rotating frame of reference indicated by the coordinates x-y-z. The geometry of the resonator is defined by the position vector $\mathbf{p} = (x_\mathrm{p}, y_\mathrm{p}, z_\mathrm{p})^\mathrm{T}$ for each mass point. Resonator vibrations are described by the displacement field $\mathbf{u}(\mathbf{p})$.

We consider a generalized gyroscope model with a three-dimensional resonator of arbitrary geometry (the green body in Supplementary Fig. **1**). This resonator is attached to a rotating reference frame x-y-z, which rotates at an angular rate $\boldsymbol{\Omega}$ relative to the inertial frame of reference x_0-y_0-z_0. By choosing the rotating frame's z-axis to align with $\boldsymbol{\Omega}$, we need only consider $\boldsymbol{\Omega}$'s z-component, maintaining generality. The mass centroid of the resonator o_p is at position vector $\mathbf{r}_1 = (x_1, y_1, z_1)^\mathrm{T}$ from the rotating frame's origin o. This rotating frame is positioned at vector $\mathbf{r}_0 = (x_0, y_0, z_0)^\mathrm{T}$ from the inertial frame's origin o_0.

Within the framework of linear elasticity theory, a vibrating resonator body possesses eigenstates, corresponding to displacement patterns of the normal modes, with eigenvalues indicating resonant frequencies. The displacement pattern of a vibrating resonator is expressed by the displacement field $\mathbf{u}(\mathbf{p})$, where $\mathbf{p} = (x_\mathrm{p}, y_\mathrm{p}, z_\mathrm{p})^\mathrm{T}$ is the position vector of resonator mass points relative to the mass centroid o_p. Considering two vibrational modes potentially coupled by the Coriolis effect, their displacement patterns are given by

$$\mathbf{u}_1(\mathbf{p}, t) = \boldsymbol{\Psi}(\mathbf{p}) q_1(t), \quad \mathbf{u}_2(\mathbf{p}, t) = \boldsymbol{\Phi}(\mathbf{p}) q_2(t). \tag{S.2}$$

Here, $\mathbf{\Psi} = (\psi_x(\mathbf{p}), \psi_y(\mathbf{p}), \psi_z(\mathbf{p}))^{\mathrm{T}}$ and $\mathbf{\Phi} = (\phi_x(\mathbf{p}), \phi_y(\mathbf{p}), \phi_z(\mathbf{p}))^{\mathrm{T}}$ represent the normalized shape functions of the modes, while q_1 and q_2 are their vibration amplitudes. The aggregate displacement field of the gyroscope resonator is a superposition of the two modes: $\mathbf{u} = \mathbf{u}_1 + \mathbf{u}_2$.

The translational motion of the rotating frame is disregarded in this work ($\dot{\mathbf{r}}_0 = \mathbf{0}$), aligning the origin o_1 of the rotating frame with that of the inertial frame o_0 ($\mathbf{r}_0 = \mathbf{0}$). The gyroscope, attached to this frame, yields $\dot{\mathbf{r}}_1 = \mathbf{0}$. The $\mathbf{r}_1$ magnitude affects only centrifugal forces and not the Coriolis effect. Thus, we place the rotating frame's origin o at the resonator's mass centroid o_{p} ($\mathbf{r}_1 = \mathbf{0}$). We consider rotation solely around the z axis with angular velocity Ω, denoted by $\mathbf{\Omega} = (0, 0, \Omega)^{\mathrm{T}}$. Consequently, the kinetic energy of the rotating and vibrating gyroscopic resonator is expressed as [4, 5]

$$\begin{aligned} T &= \frac{1}{2}\iiint\limits_V \rho\left(\dot{\mathbf{r}} + \mathbf{\Omega}\times\mathbf{r}\right)^2 \mathrm{d}V = \frac{1}{2}\iiint\limits_V \rho\left[\dot{\mathbf{u}} + \mathbf{\Omega}\times(\mathbf{p}+\mathbf{u})\right]^2 \mathrm{d}V \\ &= T_1 + T_2 + T_3, \end{aligned} \tag{S.3}$$

where $\mathbf{r} = \mathbf{r}_0 + \mathbf{r}_1 + \mathbf{p}$, ρ represents density, and

$$\begin{aligned} T_1 &= \frac{1}{2}\rho\iiint\limits_V \left(\dot{u}_x^2 + \dot{u}_y^2 + \dot{u}_z^2\right)\mathrm{d}V, \\ T_2 &= \frac{1}{2}\rho\Omega^2\iiint\limits_V \left[(x_{\mathrm{p}} + u_x)^2 + (y_{\mathrm{p}} + u_y)^2\right]\mathrm{d}V, \\ T_3 &= \rho\Omega\iiint\limits_V \left[(x_{\mathrm{p}} + u_x)\,\dot{u}_y - (y_{\mathrm{p}} + u_y)\,\dot{u}_x\right]\mathrm{d}V. \end{aligned} \tag{S.4}$$

The expansion of T_1 yields

$$\begin{aligned} T_1 = &\frac{1}{2}\dot{q}_1^2\rho\iiint\limits_V \left(\psi_x^2 + \psi_y^2 + \psi_z^2\right)\mathrm{d}V + \frac{1}{2}\dot{q}_2^2\rho\iiint\limits_V \left(\phi_x^2 + \phi_y^2 + \phi_z^2\right)\mathrm{d}V \\ &+ \dot{q}_1\dot{q}_2\rho\iiint\limits_V \left(\psi_x\phi_x + \psi_y\phi_y + \psi_z\phi_z\right)\mathrm{d}V. \end{aligned} \tag{S.5}$$

The orthogonality of normal modes causes the last term in equation (S.5) to vanish. By introducing effective inertial masses [4, 5]:

$$m_1 = \iiint\limits_V \rho\left(\psi_x^2 + \psi_y^2 + \psi_z^2\right)\mathrm{d}V, \;\; m_2 = \iiint\limits_V \rho\left(\phi_x^2 + \phi_y^2 + \phi_z^2\right)\mathrm{d}V, \tag{S.6}$$

and T_1 can be simplified to

$$T_1 = \frac{1}{2}m_1\dot{q}_1^2 + \frac{1}{2}m_2\dot{q}_2^2. \tag{S.7}$$

Evaluating T_2, we have

$$T_2 = \frac{1}{2}\Omega^2 q_1^2 \rho \iiint\limits_V \left(\psi_x^2 + \psi_y^2\right) \mathrm{d}V + \frac{1}{2}\Omega^2 q_2^2 \rho \iiint\limits_V \left(\phi_x^2 + \phi_y^2\right) \mathrm{d}V$$
$$+ \Omega^2 q_1 q_2 \rho \iiint\limits_V \left(\psi_x \phi_x + \psi_y \phi_y\right) \mathrm{d}V$$
$$+ \Omega^2 q_1 \rho \iiint\limits_V \left(x_\mathrm{p} \psi_x + y_\mathrm{p} \psi_y\right) \mathrm{d}V + \Omega^2 q_2 \rho \iiint\limits_V \left(x_\mathrm{p} \phi_x + y_\mathrm{p} \phi_y\right) \mathrm{d}V. \tag{S.8}$$

Here, T_2 relates to the centrifugal effect confined to the x-y projection of the gyroscopic resonator. Due to orthogonality of the normal modes, the third term in equation (S.8) is zero. The last two terms, which account for geometry-induced centrifugal forces on the gyroscopic modes, also nullify for bending modes. The first and second terms act as the effective centrifugal contributions to normal modes, with forces tied solely to $q_{1,2}$ amplitudes, independent of resonator geometry. Defining effective centrifugal masses [4, 5]

$$n_1 = \iiint\limits_V \rho \left(\psi_x^2 + \psi_y^2\right) \mathrm{d}V, \;\; n_2 = \iiint\limits_V \rho \left(\phi_x^2 + \phi_y^2\right) \mathrm{d}V, \tag{S.9}$$

simplifies T_2 to

$$T_2 = \frac{1}{2} n_1 \Omega^2 q_1^2 + \frac{1}{2} n_2 \Omega^2 q_2^2. \tag{S.10}$$

Reformulating T_3 while neglecting the position elements x_p and y_p, due to their lack of inertial force contribution, we have

$$T_3 = \Omega \left(q_1 \dot{q}_2 - \dot{q}_1 q_2\right) \iiint\limits_V \rho \left(\psi_x \phi_y - \psi_y \phi_x\right) \mathrm{d}V. \tag{S.11}$$

By defining the effective Coriolis mass [4, 5]

$$m_\mathrm{c} = \iiint\limits_V \rho \left(\psi_x \phi_y - \psi_y \phi_x\right) \mathrm{d}V, \tag{S.12}$$

T_3 is simplified to

$$T_3 = m_\mathrm{c} \Omega \left(q_1 \dot{q}_2 - \dot{q}_1 q_2\right). \tag{S.13}$$

The kinetic energy of the gyroscope system (S.3) can be reformulated by combining equations (S.7), (S.10), and (S.13):

$$T = \frac{1}{2} m_1 \dot{q}_1^2 + \frac{1}{2} m_2 \dot{q}_2^2 + \frac{1}{2} n_1 \Omega^2 q_1^2 + \frac{1}{2} n_2 \Omega^2 q_2^2 + m_\mathrm{c} \Omega \left(q_1 \dot{q}_2 - \dot{q}_1 q_2\right). \tag{S.14}$$

An analysis of (S.6), (S.9), and (S.12) reveals distinct definitions for inertial, centrifugal, and Coriolis masses, with the condition $m_\mathrm{c} \leq m_{1,2}$ always valid.

The *Coriolis coupling coefficient* for each mode is defined as the ratio of the Coriolis mass to the inertial mass, denoted $\kappa_{1,2} \equiv m_\mathrm{c}/m_{1,2}$. The *Coriolis interaction efficiency* is captured by the *mean Coriolis coupling coefficient* [4, 5],

$$\begin{aligned}\kappa_0 \equiv \sqrt{\kappa_1\kappa_2} &= \frac{m_\mathrm{c}}{\sqrt{m_1 m_2}} \\ &= \iiint\limits_V \frac{\psi_x\phi_y - \psi_y\phi_x}{\sqrt{(\psi_x^2+\psi_y^2+\psi_z^2)(\phi_x^2+\phi_y^2+\phi_z^2)}}\mathrm{d}V,\end{aligned} \tag{S.15}$$

termed the intrinsic Coriolis factor. It is directly determined by the mode shapes and obeys the constraint $\kappa_0 \leq 1$.

D. Hamiltonian of the Coriolis coupled system

In this analysis, we focus solely on the Coriolis coupling, with the potential energy $U(\mathbf{r})$ arising from the springs, given by

$$U(q_1, q_2) = \frac{1}{2}m_1\omega_1^2 q_1^2 + \frac{1}{2}m_2\omega_2^2 q_2^2, \tag{S.16}$$

where $\omega_{1,2}$ denote the natural frequencies of the respective modes. The stiffness coupling is excluded from this consideration.

The Lagrangian of the gyroscope resonator in a rotating reference frame, informed by equations (S.14) and (S.16), is expressed as [4, 5]:

$$\begin{aligned}L = T - U = &\frac{1}{2}m_1\dot{q}_1^2 + \frac{1}{2}m_2\dot{q}_2^2 + \frac{1}{2}\Omega^2\left(n_1 q_1^2 + n_2 q_2^2\right) + m_\mathrm{c}\Omega\left(q_1\dot{q}_2 - \dot{q}_1 q_2\right) \\ &- \frac{1}{2}m_1\omega_1^2 q_1^2 - \frac{1}{2}m_2\omega_2^2 q_2^2.\end{aligned} \tag{S.17}$$

The momenta conjugate to the displacements q_1 and q_2 are:

$$\begin{aligned}p_1 &= \frac{\partial L}{\partial \dot{q}_1} = m_1\dot{q}_1 - m_\mathrm{c}\Omega q_2, \\ p_2 &= \frac{\partial L}{\partial \dot{q}_2} = m_2\dot{q}_2 + m_\mathrm{c}\Omega q_1,\end{aligned} \tag{S.18}$$

respectively. The Hamiltonian of the rotating system is obtained from the Lagrangian (S.17) by the Legendre transform [6],

$$\begin{aligned}H = &\frac{1}{2m_1}\left(p_1 + m_\mathrm{c}\Omega q_2\right)^2 + \frac{1}{2m_2}\left(p_2 - m_\mathrm{c}\Omega q_1\right)^2 - \frac{1}{2}\Omega^2\left(n_1 q_1^2 + n_2 q_2^2\right) \\ &+ \frac{1}{2}m_1\omega_1^2 q_1^2 + \frac{1}{2}m_2\omega_2^2 q_2^2.\end{aligned} \tag{S.19}$$

The $m_c\Omega q_{1,2}$ terms act as effective magnetic fields. For our analysis, we assume the two modes are degenerate, $m = m_1 = m_2$.

We can treat this system quantum mechanically by defining the phonon annihilation operators [6]

$$b_j = \frac{1}{2q_{j,\text{ZPF}}}\left(q_j + \frac{\mathrm{i}p_j}{m\omega_j}\right), \tag{S.20}$$

for $j = 1, 2$, where $q_{j,\text{ZPF}} = [\hbar/(2m\omega_j)]^{1/2}$ represents the zero-point fluctuation amplitude of the respective mode j. In this context, the reduced Planck constant is treated as $\hbar = 1$.

The position and momentum operators for the two modes are defined as

$$\begin{aligned} q_j &= q_{j,\text{ZPF}}\left(b_j + b_j^\dagger\right), \\ p_j &= -\mathrm{i}m\omega_j q_{j,\text{ZPF}}\left(b_j - b_j^\dagger\right). \end{aligned} \tag{S.21}$$

The position and momentum operators satisfy the commutator relations $[q_j, p_j] = \mathrm{i}\hbar$. Here, $b_j^\dagger b_j$ represents the phonon number operator. Inserting these operators (S.21) into (S.19) maps the Hamiltonian into a quadratic form,

$$H = \hbar\omega_1 b_1^\dagger b_1 + \hbar\omega_2 b_2^\dagger b_2 + \frac{\mathrm{i}}{2}\hbar\eta\kappa_0\Omega\left(b_1^\dagger b_2 - b_2^\dagger b_1\right), \tag{S.22}$$

where the fast oscillating terms $b_1^\dagger b_2^\dagger$ and $b_1 b_2$ are omitted. The parameter η is approximated as $\eta = \sqrt{\omega_1/\omega_2} + \sqrt{\omega_2/\omega_1} \approx 2$ under the near-degenerate condition.

In the Heisenberg picture, the temporal evolution of the annihilation operators is governed by the Hamiltonian matrix:

$$\mathbf{H} = \begin{bmatrix} \omega_1 & \mathrm{i}\kappa_0\Omega \\ -\mathrm{i}\kappa_0\Omega & \omega_2 \end{bmatrix}. \tag{S.23}$$

The Coriolis effect is described by the *off-diagonal elements* of $\mathbf{H}$.

The eigenfrequencies (or real part of the eigenvalues) of $\mathbf{H}$ are given by

$$\lambda_\pm = \frac{\omega_1 + \omega_2}{2} \pm \sqrt{\frac{(\omega_2 - \omega_1)^2}{4} + \kappa_0^2\Omega^2}. \tag{S.24}$$

Under the degenerate condition $\omega = \omega_1 = \omega_2$, the eigenfrequencies $\lambda_\pm = \omega \pm \kappa_0\Omega$ vary linearly with angular velocity, with scale factor given by the intrinsic Coriolis factor κ_0. Subsequently, it will be demonstrated that *introducing cusp singularities in a quadrature frequency-modulated approach can amplify this linear scale factor.*

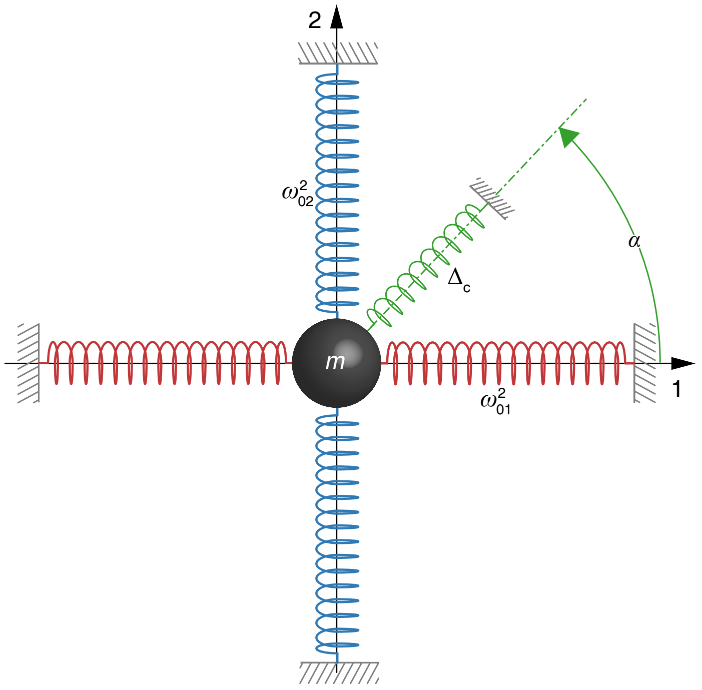

Supplementary Figure 2. **Order-reduced model of the off-axis tuning.** The to-and-fro motions of the proof mass m along the red and blue springs signify standing-wave modes 1 and 2 with natural frequencies $\omega_{1,2}$. The green spring with stiffness Δ_c centered between modes 1 and 2 can introduce stiffness coupling.

SUPPLEMENTARY NOTE 2. STIFFNESS COUPLING INTRODUCED BY OFF-AXIS TUNING

The generation of cusp singularities hinges on deploying a stiffness coupling, which is facilitated by off-axis tuning as demonstrated by the order-reduced model in Supplementary Fig. **2**. The stiffness matrix for orthogonal modes 1 and 2 is represented by diag$[m\omega_{01}^2, m\omega_{02}^2]$, where m denotes the proof inertial mass and $\omega_{01,02}$ are the initial natural frequencies of these modes. Introducing an additional off-axis spring with stiffness Δ_c (highlighted in green), aligned between the principal axes of the two normal modes, modifies the tuned stiffness matrix further,

$$\begin{aligned}\mathbf{M} &= m\begin{bmatrix}\omega_{01}^2 & 0\\ 0 & \omega_{02}^2\end{bmatrix} + \mathrm{e}^{-\mathrm{i}\alpha\sigma_2}\begin{bmatrix}\Delta_\mathrm{c} & 0\\ 0 & 0\end{bmatrix}\mathrm{e}^{\mathrm{i}\alpha\sigma_2}\\ &= \begin{bmatrix}m\omega_{01}^2 + \Delta_\mathrm{c}\cos^2\alpha & \Delta_\mathrm{c}\cos\alpha\sin\alpha\\ \Delta_\mathrm{c}\cos\alpha\sin\alpha & m\omega_{02}^2 + \Delta_\mathrm{c}\sin^2\alpha\end{bmatrix},\end{aligned} \tag{S.25}$$

where α denotes the misalignment angle of the tuning spring relative to the principal axis of mode-1. The second Pauli matrix is $\sigma_2 = \begin{bmatrix} 0 & -\mathrm{i} \\ \mathrm{i} & 0 \end{bmatrix}$. The expression

$$\exp\left(\mathrm{i}\alpha\sigma_2\right) = \begin{bmatrix} \cos\alpha & \sin\alpha \\ -\sin\alpha & \cos\alpha \end{bmatrix}$$

illustrates a rotation transformation by α.

For optimal stiffness coupling, as indicated by the off-diagonal elements in the stiffness matrix (S.25), setting α to $\pm\pi/4$ is advantageous.

SUPPLEMENTARY NOTE 3. DYNAMICS OF THE COUPLED GYROSCOPE WITH QUADRATURE DRIVE

A. Equations of motion

Now, two types of interactions are analyzed in the model: the stiffness coupling due to off-axis tuning and the Coriolis coupling from out-of-plane rotation. The potential energy for the system in Supplementary Fig. **2**, considering stiffness coupling, is expressed as [7]

$$U = \frac{1}{2}m\omega_1^2 q_1^2 + \frac{1}{2}m\omega_2^2 q_2^2 + k_\mathrm{c} q_1 q_2, \tag{S.26}$$

where $\omega_{1,2}$ are the modified mode frequencies and k_c denotes the coupling stiffness, specified by

$$\begin{aligned} \omega_1^2 &= \omega_{01}^2 + \Delta_\mathrm{c}/m\cos^2\alpha, \\ \omega_2^2 &= \omega_{02}^2 + \Delta_\mathrm{c}/m\sin^2\alpha, \\ k_\mathrm{c} &= \Delta_\mathrm{c}\cos\alpha\sin\alpha, \end{aligned} \tag{S.27}$$

accordingly. In our study, we select the initial mode frequencies to be degenerate, $\omega_0 = \omega_{01} = \omega_{02}$.

Utilizing the gyroscopic kinetic energy as described in (S.14) alongside the coupled potential energy in (S.26), the Lagrangian for the coupled gyroscope system arises as follows:

$$\begin{aligned} L = T - U = &\frac{1}{2}m\dot{q}_1^2 + \frac{1}{2}m\dot{q}_2^2 + \frac{1}{2}\Omega^2(n_1 q_1^2 + n_2 q_2^2) + m\kappa_0\Omega(q_1\dot{q}_2 - \dot{q}_1 q_2) \\ &- \frac{1}{2}m\omega_1^2 q_1^2 - \frac{1}{2}m\omega_2^2 q_2^2 - k_\mathrm{c} q_1 q_2. \end{aligned} \tag{S.28}$$

The system's Hamiltonian is obtained by performing the Legendre transformation on the Lagrangian (S.28):

$$\begin{aligned} H = &\frac{1}{2m}(p_1 + m\kappa_0\Omega q_2)^2 + \frac{1}{2m}(p_2 - m\kappa_0\Omega q_1)^2 - \frac{1}{2}\Omega^2(n_1 q_1^2 + n_2 q_2^2) \\ &+ \frac{1}{2}m\omega_1^2 q_1^2 + \frac{1}{2}m\omega_2^2 q_2^2 + k_\mathrm{c} q_1 q_2. \end{aligned} \tag{S.29}$$

The Hamiltonian canonical equations are expressed as:

$$\begin{aligned}\dot{p}_1 =& -\frac{\partial H}{\partial q_1}\\ =& -m\omega_1^2 q_1 - k_{\mathrm{c}} q_2 + \kappa_0\Omega\left(p_2 - m\kappa_0\Omega q_1\right) + n_1\Omega^2 q_1,\\ \dot{p}_2 =& -\frac{\partial H}{\partial q_2}\\ =& -m\omega_2^2 q_2 - k_{\mathrm{c}} q_1 - \kappa_0\Omega\left(p_1 + m\kappa_0\Omega q_2\right) + n_2\Omega^2 q_2.\end{aligned} \tag{S.30}$$

Substituting momenta (S.18) into (S.30) yields the Newtonian second-order differential equations governing the coupled gyroscope system's displacements $q_{1,2}$.

$$\begin{aligned}&\ddot{q}_1 + \omega_1^2 q_1 + \frac{k_{\mathrm{c}}}{m} q_2 - 2\kappa_0\Omega\dot{q}_2 - \frac{n_1}{m}\Omega^2 q_1 - \kappa_0\dot{\Omega} q_2 = 0,\\ &\ddot{q}_2 + \omega_2^2 q_2 + \frac{k_{\mathrm{c}}}{m} q_1 + 2\kappa_0\Omega\dot{q}_1 - \frac{n_2}{m}\Omega^2 q_2 + \kappa_0\dot{\Omega} q_1 = 0.\end{aligned} \tag{S.31}$$

Alternatively, we can treat the system quantum mechanically to obtain the first-order complex equations of motion. By substituting the quantum operators as defined in (S.21) into (S.29), the Hamiltonian can be expressed in a quadratic form [6, 7],

$$H = \hbar\omega_1 b_1^\dagger b_1 + \hbar\omega_2 b_2^\dagger b_2 + \mathrm{i}\hbar\kappa_0\Omega\left(b_1^\dagger b_2 - b_2^\dagger b_1\right) + \frac{1}{2}\hbar g\left(b_1^\dagger b_2 + b_2^\dagger b_1\right), \tag{S.32}$$

where the stiffness-coupling strength g is defined by

$$g = \frac{k_{\mathrm{c}}}{2m\sqrt{\omega_1\omega_2}} \approx \frac{\Delta_{\mathrm{c}}\cos\alpha\sin\alpha}{2m\omega_0}. \tag{S.33}$$

The system's temporal evolution is characterized by the Heisenberg-Langevin equations for the creation operators $b_j^\dagger$,

$$\frac{\mathrm{d}}{\mathrm{d}t} b_j^\dagger = \frac{\mathrm{i}}{\hbar}[H, b_j^\dagger] - \frac{\gamma}{2} b_j^\dagger + \xi_j. \tag{S.34}$$

Here, γ represents the dissipation rate of the operational modes, while ξ_j denotes the thermal Langevin force exerted on the mode $b_j^\dagger$. We focus solely on coherent states and ignore the fluctuation term. We define the complex variables $a_j \equiv \langle b_j^\dagger \rangle$ to obtain the first-order complex differential equations of motion [8],

$$\begin{bmatrix}\dot{a}_1\\ \dot{a}_2\end{bmatrix} = \begin{bmatrix}\mathrm{i}\omega_1 - \gamma/2 & \mathrm{i}g/2 + \kappa_0\Omega\\ \mathrm{i}g/2 - \kappa_0\Omega & \mathrm{i}\omega_2 - \gamma/2\end{bmatrix}\begin{bmatrix}a_1\\ a_2\end{bmatrix}. \tag{S.35}$$

Notably, by employing the rotating wave approximation [9], the second-order real differential equations of motion (S.31) are equivalent to the first-order complex differential equations of motion (S.35). The relationship between the variables is given by

$$q_j = \left(a_j + a_j^*\right)/\sqrt{2m\omega_j}. \tag{S.36}$$

In the following analyses, (S.35) are used for simplicity.

B. Steady-states from the quadrature drive

We consider the quadrature drive condition: Simultaneously actuate modes 1 and 2 with two equi-strength sinusoidal forces $F_1 = F\cos(\omega_\mathrm{d} t)$ and $F_2 = F\cos(\omega_\mathrm{d} t + \pi/2)$ in quadrature, respectively. The inhomogeneous equations of motion are given by

$$\begin{bmatrix} \dot{a}_1 \\ \dot{a}_2 \end{bmatrix} = \begin{bmatrix} \mathrm{i}\omega_1 - \gamma/2 & \mathrm{i}g/2 + \kappa_0\Omega \\ \mathrm{i}g/2 - \kappa_0\Omega & \mathrm{i}\omega_2 - \gamma/2 \end{bmatrix} \begin{bmatrix} a_1 \\ a_2 \end{bmatrix} - \begin{bmatrix} \mathrm{i} \\ -1 \end{bmatrix} f\mathrm{e}^{\mathrm{i}\omega_\mathrm{d} t}, \tag{S.37}$$

with $f \approx \frac{1}{2}F(2m\omega_0)^{-1/2}$.

To derive the steady states, we convert the system to a frame rotating at the driving frequency ω_d by writing $a_j = A_j \mathrm{e}^{\mathrm{i}\omega_\mathrm{d} t}$. In the rotating frame, the equations of motion for the complex amplitudes A_j are described by

$$\begin{bmatrix} \dot{A}_1 \\ \dot{A}_2 \end{bmatrix} = \begin{bmatrix} -\mathrm{i}\Delta_1 - \gamma/2 & \mathrm{i}g/2 + \kappa_0\Omega \\ \mathrm{i}g/2 - \kappa_0\Omega & -\mathrm{i}\Delta_2 - \gamma/2 \end{bmatrix} \begin{bmatrix} A_1 \\ A_2 \end{bmatrix} - \begin{bmatrix} \mathrm{i} \\ -1 \end{bmatrix} f, \tag{S.38}$$

where $\Delta_j \equiv \omega_\mathrm{d} - \omega_j$ represents the drive detunings. The steady-state solutions are derived by setting $\dot{A}_j = 0$, resulting in $A_j = f\chi_j(\omega_\mathrm{d})$. For modes $j = 1, 2$, the mechanical susceptibilities χ_j are expressed as

$$\begin{aligned} \chi_1(\omega_\mathrm{d}) &= \frac{\omega_2 - \kappa_0\Omega - \omega_\mathrm{d} + \mathrm{i}(\gamma - g)/2}{(\omega_\mathrm{d} - \omega_+ - \mathrm{i}\gamma/2)(\omega_\mathrm{d} - \omega_- - \mathrm{i}\gamma/2)}, \\ \chi_2(\omega_\mathrm{d}) &= \frac{\mathrm{i}(\omega_1 - \kappa_0\Omega - \omega_\mathrm{d}) - (\gamma + g)/2}{(\omega_\mathrm{d} - \omega_+ - \mathrm{i}\gamma/2)(\omega_\mathrm{d} - \omega_- - \mathrm{i}\gamma/2)}, \end{aligned} \tag{S.39}$$

where $\omega_\pm$ are the eigenfrequencies of the system, given by

$$\omega_\pm = \omega \pm \sqrt{\kappa_0^2\Omega^2 + g^2/4 + \Delta\omega^2/4}, \tag{S.40}$$

where $\omega = (\omega_1 + \omega_2)/2$ represents the mean frequency and $\Delta\omega = \omega_2 - \omega_1$ denotes the difference in natural frequency between the operational modes.

According to the variable relationship (S.36), the observable steady-state mechanical displacements for modes $j = 1, 2$ are expressed as

$$q_j(\omega_\mathrm{d}, t) = [\chi_j(\omega_\mathrm{d})\mathrm{e}^{\mathrm{i}\omega_\mathrm{d} t} + \text{c.c.}]F/(4m\omega_0).$$

By writing $q_j = |q_j|\cos(\omega_\mathrm{d} t + \theta_j)$ we obtain the frequency responses of the amplitudes $|q_j|$ and phases θ_j,

$$\begin{aligned} |q_j(\omega_\mathrm{d})| &= \frac{F}{2m\omega_0}|\chi_j(\omega_\mathrm{d})|, \\ \theta_j(\omega_\mathrm{d}) &= \mathrm{Arg}[\chi_j(\omega_\mathrm{d})], \end{aligned} \tag{S.41}$$

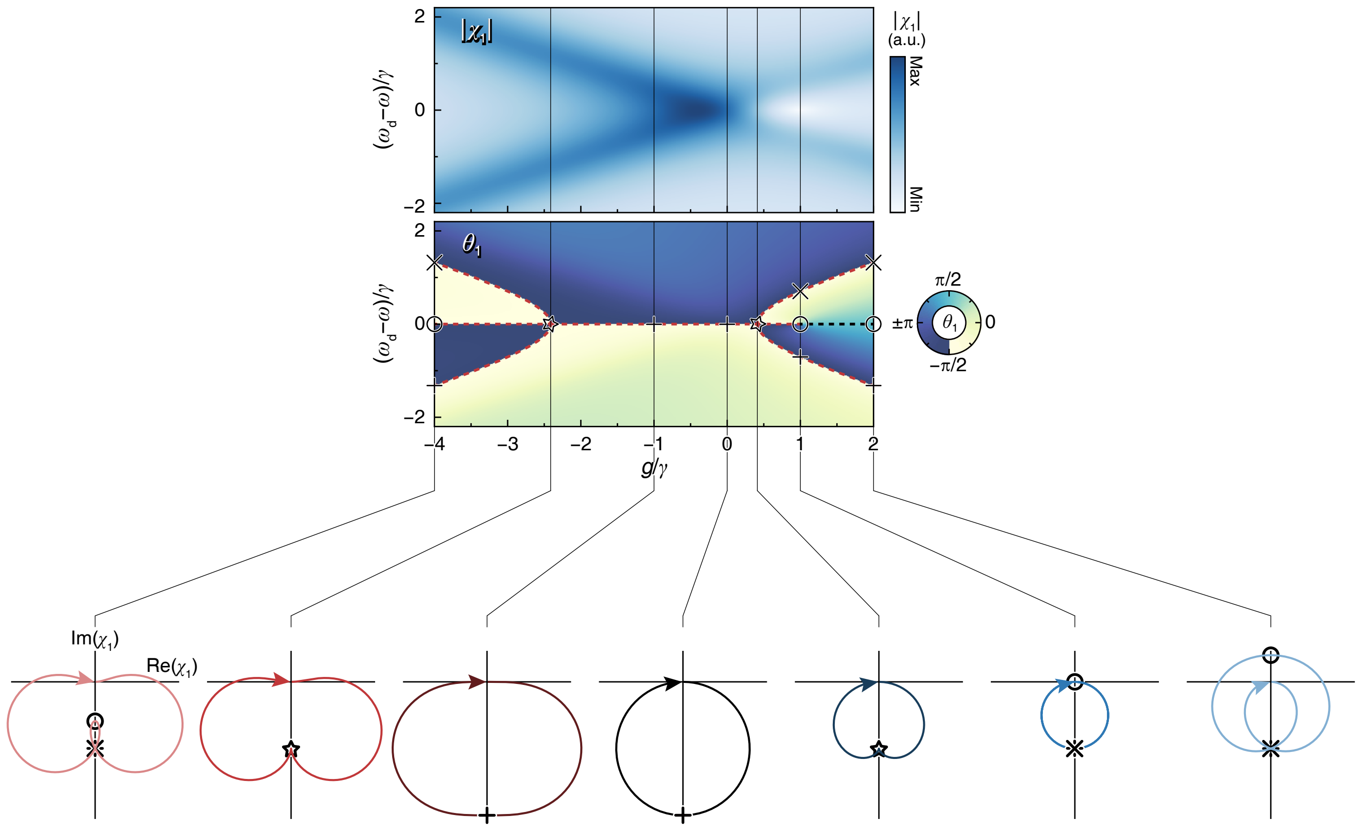

Supplementary Figure 3. **Polar plots of the mode-1 responses for $\Omega = 0$ and $g = \{-4, -\sqrt{2} - 1, -1, 0, \sqrt{2} - 1, 1, 2\}\gamma$.** The horizontal and vertical axes of the polar plots denote the real and imaginary components of the mode-1 susceptibility $\chi_1(\omega_\mathrm{d})$, respectively. The radial distance from any point on the plot to the origin indicates the normalized mode-1 amplitude $|\chi_1|$, while the angle in the complex plane corresponds to the phase θ_1. The arrow within the plot shows the direction in which ω_d increases.

where Arg denotes the argument of a complex number.

Numerical simulations of the frequency responses $|q_{1,2}(\omega_\mathrm{d})|$ and $\theta_{1,2}(\omega_\mathrm{d})$ under $g = 0$, $(-\sqrt{2} - 1)\gamma$, $(\sqrt{2} - 1)\gamma$, and $\Omega = 0$ are shown in Extended Data Fig. 4. The calculations were based on a mode frequency $\omega_0 \approx 2\pi \times 40.4$ kHz, a damping rate $\gamma = 2\pi \times 0.36$ Hz, and a Coriolis factor $\kappa_0 = 0.588$.

C. Phase-tracked closed-loop oscillation

The phase-tracked (PhT) states are those steady states whose θ_1 responses are tracked to a quadrature phase. Supplementary Figure **3** presents polar plots for the mode-1 responses at $\Omega = 0$ and $g = \{-4, -\sqrt{2} - 1, -1, 0, \sqrt{2} - 1, 1, 2\}\gamma$. In these plots, the horizontal and vertical axes denote the real and imaginary components of the mode-1 susceptibility χ_1, respectively. The radial distance from any point on the plot to

the origin indicates the normalized amplitude of mode 1, while the angle in the complex plane corresponds to the phase θ_1. The arrow within the plot shows the direction in which ω_d increases.

At $g = \gamma$, there is a phase singularity θ_1, denoted as PS_1, which enables a *topological phase transition*. In the trivial topological phase where $g < \gamma$, polar plots do not encircle the origin, and θ_1 shows no net accumulation as ω_d changes from well below to well above ω. Conversely, in the nontrivial topological phase where $g > \gamma$, polar plots encircle the origin once, and θ_1 accumulates a -2π shift if ω_d traverses from far below to far above ω.

The $\theta_1 = -\pi/2$ phase-tracked steady states appear at the intersection of polar plots and the negative half of the imaginary axis in Supplementary Fig. **3**. The oscillation frequency ω_T corresponding to the $\theta_1 = -\pi/2$ phase-tracked condition is determined by setting the real part of the mode-1 susceptibility to zero, i.e., $\mathrm{Re}[\chi_1(\omega_\mathrm{d})] = 0$, leading to the equation:

$$-(\omega_\mathrm{T} - \omega)^3 - \left(\kappa_0\Omega - \frac{\Delta\omega}{2}\right)(\omega_\mathrm{T} - \omega)^2 - \left(\frac{\gamma^2}{4} - \frac{g\gamma}{2} - \frac{g^2}{4} - \kappa_0^2\Omega^2 - \frac{\Delta\omega^2}{4}\right)(\omega_\mathrm{T} - \omega) - \left(\frac{\Delta\omega}{2} - \kappa_0\Omega\right)\left(\frac{\gamma^2}{4} + \frac{g^2}{4} + \kappa_0^2\Omega^2 + \frac{\Delta\omega^2}{4}\right) = 0. \quad \text{(S.42)}$$

This is the governing equation of the phase-tracked frequency ω_T.

In the *nontrivial topological phase* ($g > \gamma$), the polar plot also reaches the positive half-imaginary axis, which relates to $\theta_1 = \pi/2$, a condition irrelevant to this study. Thus, it is necessary to incorporate the condition $\mathrm{Im}[\chi_1(\omega_\mathrm{d})] < 0$ alongside the phase-tracked equation (S.42),

$$\frac{g+\gamma}{2}(\omega_\mathrm{T} - \omega)^2 + \gamma\left(\kappa_0\Omega - \frac{\Delta\omega}{2}\right)(\omega_\mathrm{T} - \omega) + \frac{\gamma - g}{2}\left(\frac{\gamma^2}{4} + \frac{g^2}{4} + \kappa_0^2\Omega^2 + \frac{\Delta\omega^2}{4}\right) > 0. \quad \text{(S.43)}$$

The folded surface presented in Fig. 1d of the main text is derived from conditions (S.42) and (S.43).

SUPPLEMENTARY NOTE 4. STABILITY ANALYSIS

This section investigates the phase-tracked state stability by examining the Jacobian matrix arising from the dynamical equations. Beginning with the equations of motion (S.38) within the rotating frame, we decompose the complex variables into real components: $A_{1,2} = f|\chi_{1,2}|\mathrm{e}^{\mathrm{i}\theta_{1,2}}$, where $|\chi_{1,2}|, \theta_{1,2} \in \mathbb{R}$ are the normalized amplitudes and phases. The complex equations of motion (S.38) are thus translated into four real differential equations.

$$\frac{\mathrm{d}}{\mathrm{d}t}|\chi_1| = -\frac{\gamma}{2}|\chi_1| + \kappa_0\Omega|\chi_2|\cos(\theta_2-\theta_1) - \frac{g}{2}|\chi_2|\sin(\theta_2-\theta_1) - \sin\theta_1, \tag{S.44}$$
$$\frac{\mathrm{d}}{\mathrm{d}t}|\chi_2| = -\frac{\gamma}{2}|\chi_2| - \kappa_0\Omega|\chi_1|\cos(\theta_2-\theta_1) + \frac{g}{2}|\chi_1|\sin(\theta_2-\theta_1) + \cos\theta_2, \tag{S.45}$$
$$\frac{\mathrm{d}}{\mathrm{d}t}\theta_1 = -(\omega_\mathrm{d}-\omega_1)|\chi_1| + \frac{g}{2}|\chi_2|\cos(\theta_2-\theta_1) + \kappa_0\Omega|\chi_2|\sin(\theta_2-\theta_1) - \cos\theta_1, \tag{S.46}$$
$$\frac{\mathrm{d}}{\mathrm{d}t}\theta_2 = -(\omega_\mathrm{d}-\omega_2)|\chi_2| + \frac{g}{2}|\chi_1|\cos(\theta_2-\theta_1) + \kappa_0\Omega|\chi_1|\sin(\theta_2-\theta_1) - \sin\theta_2. \tag{S.47}$$

Applying the phase-tracking condition $\theta_1 = -\pi/2$, $\mathrm{d}\theta_1/\mathrm{d}t = 0$, and $\omega_\mathrm{d} = \omega_\mathrm{T}$ to the preceding equations of motion, results in equation (S.46) transforming into the phase-tracked governing equation (S.42). Treating phase tracking control as a dynamic process with respect to the phase-tracked frequency ω_T, we derive the following phase-tracked equations of motion:

$$\frac{\mathrm{d}}{\mathrm{d}t}|\chi_1| = 1 - \frac{\gamma}{2}|\chi_1| - \kappa_0\Omega|\chi_2|\sin\theta_2 - \frac{g}{2}|\chi_2|\cos\theta_2, \tag{S.48}$$
$$\frac{\mathrm{d}}{\mathrm{d}t}|\chi_2| = \kappa_0\Omega|\chi_1|\sin\theta_2 + \frac{g}{2}|\chi_1|\cos\theta_2 - \frac{\gamma}{2}|\chi_2| + \cos\theta_2, \tag{S.49}$$
$$\frac{\mathrm{d}}{\mathrm{d}t}\theta_2 = \kappa_0\Omega|\chi_1|\cos\theta_2 - \frac{g}{2}|\chi_1|\sin\theta_2 - (\omega_\mathrm{T}-\omega_2)|\chi_2| - \sin\theta_2, \tag{S.50}$$
$$\begin{aligned}\frac{\mathrm{d}}{\mathrm{d}t}\omega_\mathrm{T} = &-(\omega_\mathrm{T}-\omega)^3 - \left(\kappa_0\Omega - \frac{\Delta\omega}{2}\right)(\omega_\mathrm{T}-\omega)^2 \\ &- \left(\frac{\gamma^2}{4} - \frac{g\gamma}{2} - \frac{g^2}{4} - \kappa_0^2\Omega^2 - \frac{\Delta\omega^2}{4}\right)(\omega_\mathrm{T}-\omega) \\ &- \left(\frac{\Delta\omega}{2} - \kappa_0\Omega\right)\left(\frac{\gamma^2}{4} + \frac{g^2}{4} + \kappa_0^2\Omega^2 + \frac{\Delta\omega^2}{4}\right).\end{aligned} \tag{S.51}$$

The phase-tracked steady-state oscillations are derived by setting $\mathrm{d}|\chi_1|/\mathrm{d}t = \mathrm{d}|\chi_2|/\mathrm{d}t = \mathrm{d}\theta_2/\mathrm{d}t = \mathrm{d}\omega_\mathrm{T}/\mathrm{d}t = 0$, confirming prior results from the rotating-wave approximation.

The Jacobian matrix for the dynamical equations (S.48)-(S.51) is denoted as $\mathbf{J}_{4\times4}$, with its components

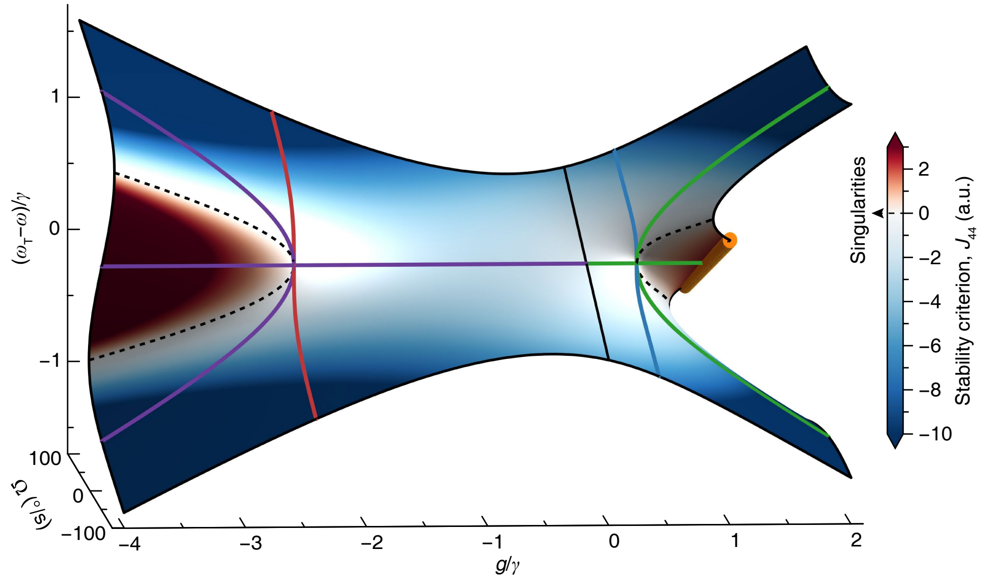

Supplementary Figure 4. **Stability analysis.** Stability in the phase-tracked steady states is determined by the Jacobian element J_{44}, which is represented by the color gradients of the ω_T surface. A positive J_{44} denotes divergence and instability, whereas a negative J_{44} signifies convergence and stability in phase-tracking control. The black dashed curves depict the critical stability limits.

defined by

$$\begin{aligned}
&J_{11} = -\frac{\gamma}{2}, \quad J_{12} = \frac{\gamma}{2}\frac{|\chi_1|}{|\chi_2|} - \frac{1}{|\chi_2|}, \quad J_{13} = (-\omega_T + \omega_1)|\chi_1|, \quad J_{14} = 0, \\
&J_{21} = -\frac{\gamma}{2}\frac{|\chi_1|}{|\chi_2|} + \frac{1}{|\chi_2|}, \quad J_{22} = -\frac{\gamma}{2}, \quad J_{23} = (\omega_T - \omega_2)|\chi_2|, \quad J_{24} = 0, \\
&J_{31} = (\omega_T - \omega_1)\frac{|\chi_1|}{|\chi_2|}, \quad J_{32} = (-\omega_T + \omega_2), \quad J_{33} = -\frac{\gamma}{2}|\chi_2|, \quad J_{34} = -|\chi_2|, \\
&J_{41} = 0, \quad J_{42} = 0, \quad J_{43} = 0, \\
&J_{44} = -3(\omega_T - \omega)^2 - (2\kappa_0\Omega - \Delta\omega)(\omega_T - \omega) - \frac{\gamma^2}{4} + \frac{g\gamma}{2} + \frac{g^2}{4} + \kappa_0^2\Omega^2 + \frac{\Delta\omega^2}{4},
\end{aligned} \tag{S.52}$$

where

$$\begin{aligned}
|\chi_1| &= \left\{ \frac{(\omega_2 - \kappa_0\Omega - \omega_T)^2 + (\gamma - g)^2/4}{[(\omega_T - \omega)^2 - \kappa_0^2\Omega^2 - g^2/4 - \gamma^2/4 - \Delta\omega^2/4]^2 + \gamma^2(\omega - \omega_T)^2} \right\}^{1/2}, \\
|\chi_2| &= \left\{ \frac{(\gamma + g)^2/4 + (\omega_1 - \kappa_0\Omega - \omega_T)^2}{[(\omega_T - \omega)^2 - \kappa_0^2\Omega^2 - g^2/4 - \gamma^2/4 - \Delta\omega^2/4]^2 + \gamma^2(\omega - \omega_T)^2} \right\}^{1/2}.
\end{aligned} \tag{S.53}$$

The stability of the phase-tracked steady states is evaluated by examining the real parts of the eigenvalues of the Jacobian matrix $\mathbf{J}$, which indicate the system's behavior around fixed points when small disturbances occur [10]. If all the real parts are negative, the system is convergent near the fixed point, and the phase-

tracked steady state is stable. Conversely, a non-negative real part in any eigenvalue implies the phase-tracked steady state is unstable or, if zero, critically stable, the latter defined as singularities.

The real components of the initial three eigenvalues of **J** consistently remain negative. Thus, the overall stability hinges on the final eigenvalue, precisely given by J_{44}, primarily dictated by the phase-control dynamics.

Supplementary Figure **4** illustrates the computed J_{44} values for phase-tracked steady states at various phase-tracked frequencies under different control parameters. In regions of inflection, positive J_{44} values suggest instability of these steady states. Black dashed curves denote the critical stability points, representing the cusp catastrophes.

SUPPLEMENTARY NOTE 5. CUSP CATASTROPHE

The critical stability limits determine the catastrophe locations [11, 12], identified by $J_{44} = 0$. To derive the catastrophe formula, we begin by reformulating the governing equation for the phase-tracked frequency (S.42) as

$$A(\omega_{\mathrm{T}} - \omega)^3 + B(\omega_{\mathrm{T}} - \omega)^2 + C(\omega_{\mathrm{T}} - \omega) + D = 0, \tag{S.54}$$

where

$$\begin{aligned}
A &= -1, \\
B &= -(\kappa_0\Omega - \frac{\Delta\omega}{2}), \\
C &= -\left(\frac{\gamma^2}{4} - \frac{g\gamma}{2} - \frac{g^2}{4} - \kappa_0^2\Omega^2 - \frac{\Delta\omega^2}{4}\right), \\
D &= -\left(\frac{\Delta\omega}{2} - \kappa_0\Omega\right)\left(\frac{\gamma^2}{4} + \frac{g^2}{4} + \kappa_0^2\Omega^2 + \frac{\Delta\omega^2}{4}\right).
\end{aligned} \tag{S.55}$$

By defining a new variable

$$Y = \omega_{\mathrm{T}} - \omega + \frac{B}{3A}, \tag{S.56}$$

the governing equation (S.54) can be simplified as

$$Y^3 + P_1 Y + P_0 = 0, \tag{S.57}$$

where the parameters $P_{0,1}$ are defined by

$$\begin{aligned}
P_1 &= \frac{3AC - B^2}{3A^2}, \\
P_0 &= \frac{2B^3 - 9ABC + 27A^2 D}{27A^3}.
\end{aligned} \tag{S.58}$$

Therefore,

$$J_{44} = \frac{\partial}{\partial Y}(Y^3 + P_1 Y + P_0) = 3Y^2 + P_1. \tag{S.59}$$

Catastrophes can be derived by integrating $J_{44} = 0$ with the phase-tracked frequency governing equation

$$\begin{cases} 3Y^2 + P_1 = 0 \\ Y^3 + P_1 Y + P_0 = 0 \end{cases} \tag{S.60}$$

Projected to the g-Ω plane, the catastrophes are given by

$$\frac{P_0^2}{4} + \frac{P_1^3}{27} = 0. \tag{S.61}$$

SUPPLEMENTARY NOTE 6. STATE INFORMATION

Understanding the state information linked to the phase-tracked singularities is vital for grasping its complete dynamics. The hybrid phase-tracked oscillation state is denoted by the state vector $|\psi\rangle = a_1|1\rangle + a_2|2\rangle$, where $a_{1,2} = f\chi_{1,2}(\omega_\mathrm{T})\mathrm{e}^{\mathrm{i}\omega_\mathrm{d}t}$ represent the complex amplitudes for modes 1 and 2. The vectors $|1\rangle = (1,0)^\mathrm{T}$ and $|2\rangle = (0,1)^\mathrm{T}$, corresponding to modes 1 and 2, respectively, compose the orthonormal standing-wave basis $\{|1\rangle, |2\rangle\}$. With the mechanical susceptibilities $\chi_{1,2}(\omega_\mathrm{T})$ for the phase-tracked steady states expressed by (S.39), substituting these into $|\psi\rangle$ leads to further expression as

$$|\psi\rangle = f\sqrt{|\chi_1|^2 + |\chi_2|^2}\mathrm{e}^{\mathrm{i}(\omega_\mathrm{d}t+\theta_1)}\left(\cos\frac{\phi}{2}|1\rangle + \mathrm{e}^{\mathrm{i}\vartheta}\sin\frac{\phi}{2}|2\rangle\right), \tag{S.62}$$

where $\phi \in [0, \pi]$ is the polar angle defined by

$$\begin{aligned} \phi &\equiv 2\arctan\frac{|\chi_2|}{|\chi_1|} \ \text{ or } \ 2\arctan\frac{|q_2|}{|q_1|} \\ &= 2\arctan\sqrt{\frac{(\omega_\mathrm{T} - \omega + \kappa_0\Omega + \Delta\omega/2)^2 + (\gamma + g)^2/4}{(-\omega_\mathrm{T} + \omega - \kappa_0\Omega + \Delta\omega/2)^2 + (\gamma - g)^2/4}}, \end{aligned} \tag{S.63}$$

and the azimuthal angle $\vartheta \in [-\pi, \pi)$ is the relative phase of the standing-wave modes

$$\begin{aligned} \vartheta &\equiv \theta_2 - \theta_1 = \mathrm{Arg}\left[\frac{\chi_2(\omega_\mathrm{T})}{\chi_1(\omega_\mathrm{T})}\right] \\ &= \mathrm{Arg}\left\{g(\omega_\mathrm{T} - \omega + \kappa_0\Omega) - \frac{\Delta\omega\gamma}{2} + \mathrm{i}\left[(\omega_\mathrm{T} - \omega + \kappa_0\Omega)^2 - \frac{\Delta\omega^2}{4} + \frac{\gamma^2}{4} - \frac{g^2}{4}\right]\right\}. \end{aligned} \tag{S.64}$$

Due to the gauge invariance of the $\theta_1 = -\pi/2$ phase-tracked dynamics, we can disregard the global phase in (S.62) and utilize the normalized state vector in the subsequent analyses,

$$|\psi\rangle = \cos\frac{\phi}{2}|1\rangle + \mathrm{e}^{\mathrm{i}\vartheta}\sin\frac{\phi}{2}|2\rangle \tag{S.65}$$

to describe the phase-tracked hybridized states.

Each state vector represents a polarization pattern of the effective proof mass in the q_1-q_2 plane, depicted in Fig. 1a. For state visualization, the normalized state vector is projected onto a Poincaré (or classic Bloch) sphere, as illustrated in Fig. 1e. The state vectors in spherical coordinates are $(S_1, S_2, S_3)^{\mathrm{T}}$, defined by

$$\begin{aligned}
S_1 &= \sin\phi\cos\vartheta = 2\frac{\mathrm{Re}(\chi_1)\mathrm{Re}(\chi_2) + \mathrm{Im}(\chi_1)\mathrm{Im}(\chi_2)}{|\chi_1|^2 + |\chi_2|^2} \\
&= \frac{(\omega_{\mathrm{T}} - \omega + \kappa_0\Omega)g - \Delta\omega\gamma/2}{(\omega_{\mathrm{T}} - \omega + \kappa_0\Omega)^2 + \Delta\omega^2/4 + \gamma^2/4 + g^2/4}, \\
S_2 &= \sin\phi\sin\vartheta = 2\frac{\mathrm{Re}(\chi_1)\mathrm{Im}(\chi_2) - \mathrm{Im}(\chi_1)\mathrm{Re}(\chi_2)}{|\chi_1|^2 + |\chi_2|^2} \\
&= \frac{(\omega_{\mathrm{T}} - \omega + \kappa_0\Omega)^2 - \Delta\omega^2/4 + \gamma^2/4 - g^2/4}{(\omega_{\mathrm{T}} - \omega + \kappa_0\Omega)^2 + \Delta\omega^2/4 + \gamma^2/4 + g^2/4}, \\
S_3 &= \cos\phi = \frac{|\chi_1|^2 - |\chi_2|^2}{|\chi_1|^2 + |\chi_2|^2} \\
&= \frac{-(\omega_{\mathrm{T}} - \omega + \kappa_0\Omega)\Delta\omega - g\gamma/2}{(\omega_{\mathrm{T}} - \omega + \kappa_0\Omega)^2 + \Delta\omega^2/4 + \gamma^2/4 + g^2/4}.
\end{aligned} \tag{S.66}$$

The ellipticity S_1 signifies polarization orbits that are circular, elliptical, or linear for conditions $|S_1| = 0$, $0 < |S_1| < 1$, and $|S_1| = 1$, correspondingly. The chirality S_2 indicates CW or CCW polarization orbits when $S_2 > 0$ or $S_2 < 0$, respectively. The polarization orientation S_3 reflects the relative population of the states $|1\rangle$ and $|2\rangle$.

In the scenario where standing-wave modes are fully degenerate ($\Delta\omega = 0$) and there is no rotation or coupling ($\Omega = 0$, $g = 0$), a quadrature drive creates a CW mode positioned at $(0, 1, 0)$ on the Poincaré sphere, the operational point for the quadrature frequency-modulated (QFM) gyroscope. Increasing the coupling strength g from zero to $(\sqrt{2}-1)\gamma$ propels the evolution of the system along a green path in Fig. 1e from $(0, 1, 0)$ to X_1 at $(0, 1/\sqrt{2}, -1/\sqrt{2})$, representing a vertically directed elliptical CW polarization. With further increments in g, the system abruptly and randomly shifts to one of the two stable states, eventually reaching $(\pm1, 0, 0)$ as $g \to \infty$. The central unstable path concludes at $(0, 0, -1)$ when $g = \gamma$, marking a θ_1 phase singularity PS_1 with a topological charge of -1 (Fig. 1c), stemming from destructive interference between the two traveling-wave modes.

When the coupling strength g is reduced from zero, the system follows the purple path in Fig. 1e. Initially, it reaches $(0, 0, 1)$ at $g = -\gamma$, indicating a phase singularity PS_2 with a topological charge of $+1$ in the mode-2 phase θ_2 or the relative phase ϑ, marked by the black square in Fig. 1d. As g decreases further to $(-\sqrt{2}-1)\gamma$, the system progresses to X_2 at $(0, -1/\sqrt{2}, 1/\sqrt{2})$, which is linked to a horizontally oriented elliptical CCW polarization. With a continued decrease in g, the system randomly jumps to one of the two stable branches, eventually evolving to $(\pm1, 0, 0)$ as g approaches $-\infty$. The central unstable branch

transitions to $(0, -1, 0)$ for $g \to -\infty$.

We change the angular velocity Ω while maintaining $g = (\sqrt{2} - 1)\gamma$ or $(-\sqrt{2} - 1)\gamma$. The system will then follow the blue or red paths in Fig. 1e, respectively, with arrows indicating the direction of increasing Ω. As $|\Omega| \to -\infty$, the system tends toward $(0, 1, 0)$. Varying Ω from $-\infty$ to ∞ results in the state vector on the Poincaré sphere tracing a loop that either encloses or excludes the origin of the S_1-S_2 plane, depending on whether $g = (-\sqrt{2} - 1)\gamma$ or $(\sqrt{2} - 1)\gamma$.

SUPPLEMENTARY NOTE 7. DESCRIPTION IN TRAVELING-WAVE BASIS

Previous investigations employed the standing-wave (SW) basis $\{|1\rangle, |2\rangle\}$ to describe the phase-tracked dynamics. This analysis will now examine the system using the traveling-wave (TW) basis $\{|\mathrm{CW}\rangle, |\mathrm{CCW}\rangle\}$, providing better insight into the operation, where

$$\begin{aligned} |\mathrm{CW}\rangle &= \frac{1}{\sqrt{2}}(|1\rangle + \mathrm{i}|2\rangle), \\ |\mathrm{CCW}\rangle &= \frac{1}{\sqrt{2}}(|1\rangle - \mathrm{i}|2\rangle). \end{aligned} \tag{S.67}$$

A state vector can be expressed in either basis: $|\psi\rangle = a_1|1\rangle + a_2|2\rangle = c_{\circlearrowright}|\mathrm{CW}\rangle + c_{\circlearrowleft}|\mathrm{CCW}\rangle$, where

$$\begin{aligned} a_1 &= \langle 1|\psi\rangle, \\ a_2 &= \langle 2|\psi\rangle, \end{aligned} \tag{S.68}$$

are complex amplitudes of the standing-wave modes and

$$\begin{aligned} c_{\circlearrowright} &= \langle \mathrm{CW}|\psi\rangle, \\ c_{\circlearrowleft} &= \langle \mathrm{CCW}|\psi\rangle, \end{aligned} \tag{S.69}$$

denoting those of the traveling-wave modes. The vectors from the two bases can be transformed using a unitary operator

$$\mathbf{U} = \frac{1}{\sqrt{2}} \begin{bmatrix} 1 & -\mathrm{i} \\ 1 & \mathrm{i} \end{bmatrix}, \tag{S.70}$$

such that

$$\begin{bmatrix} c_{\circlearrowright} \\ c_{\circlearrowleft} \end{bmatrix} = \mathbf{U} \begin{bmatrix} a_1 \\ a_2 \end{bmatrix}. \tag{S.71}$$

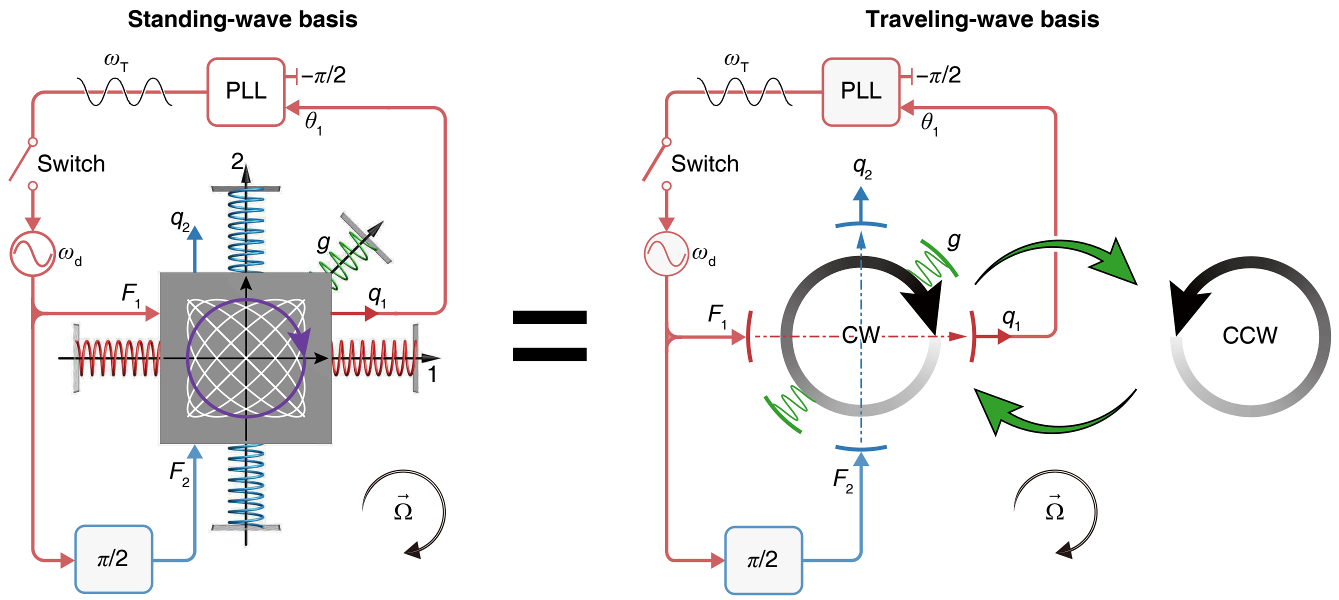

Supplementary Figure 5. The equivalent conceptual schematics of the coupled gyroscope in standing-wave and traveling-wave bases. PLL, phase-locked loop. Modes 1 and 2 are standing-wave modes, and modes CW and CCW are traveling-wave modes.

With the application of $\mathbf{U}$, the equations of motion (S.37) in the standing-wave basis are converted to the traveling-wave basis,

$$\begin{aligned}\begin{bmatrix}\dot{c}_{\circlearrowright}\\ \dot{c}_{\circlearrowleft}\end{bmatrix} &= \mathbf{U}\begin{bmatrix}\mathrm{i}\omega_1-\gamma/2 & \mathrm{i}g/2+\kappa_0\Omega\\ \mathrm{i}g/2-\kappa_0\Omega & \mathrm{i}\omega_2-\gamma/2\end{bmatrix}\mathbf{U}^{-1}\begin{bmatrix}c_{\circlearrowright}\\ c_{\circlearrowleft}\end{bmatrix}-\mathbf{U}\begin{bmatrix}\mathrm{i}\\ -1\end{bmatrix}f\mathrm{e}^{\mathrm{i}\omega_{\mathrm{d}}t}\\ &= \begin{bmatrix}\mathrm{i}(\omega+\kappa_0\Omega)-\gamma/2 & g/2-\mathrm{i}\Delta\omega/2\\ -g/2-\mathrm{i}\Delta\omega/2 & \mathrm{i}(\omega-\kappa_0\Omega)-\gamma/2\end{bmatrix}\begin{bmatrix}c_{\circlearrowright}\\ c_{\circlearrowleft}\end{bmatrix}-\begin{bmatrix}\sqrt{2}\mathrm{i}\\ 0\end{bmatrix}f\mathrm{e}^{\mathrm{i}\omega_{\mathrm{d}}t}.\end{aligned} \tag{S.72}$$

Within the traveling-wave framework, rotation causes the Coriolis effect to shift the frequency of the CW or CCW modes by $\pm\kappa_0\Omega$, akin to the *rotational Doppler effect* [13, 14]. In the traveling-wave basis, the stiffness interaction continues to facilitate coherent coupling. The frequency difference $\Delta\omega$ between the standing-wave modes translates to a stiffness coupling for the traveling-wave modes. A quadrature drive on standing-wave modes 1 and 2 yields an effective torque that stimulates either the CW or CCW traveling-wave mode (in this analysis, the CW mode is engaged with a $\pi/2$ phase shift in F_2). Assuming the degenerate scenario, $\Delta\omega = 0$, Supplementary Figure **5** shows the coupled gyroscope schematics for both standing-wave and traveling-wave bases.

SUPPLEMENTARY NOTE 8. BOOSTED FREQUENCY MODULATION NEAR CUSP-SINGULARITIES

The sharp transitions in phase-tracked frequency ω_{T} near cusp singularities $X_{1,2}$ suggest that even minor perturbations in control parameters can dramatically affect ω_{T}.

Now, we examine perturbations in the angular velocity Ω. The output for phase-tracked frequency near points X_1 or X_2, as functions of Ω, is derived by substituting $g = (\sqrt{2}-1)\gamma$ or $(-\sqrt{2}-1)\gamma$ into the governing equation for ω_{T} (S.42), respectively, as follows

$$\begin{aligned}(\omega_{\mathrm{T}}-\omega)^3 &+ \left(\kappa_0\Omega - \frac{\Delta\omega}{2}\right)(\omega_{\mathrm{T}}-\omega)^2 - \left(\kappa_0^2\Omega^2 + \frac{\Delta\omega^2}{4}\right)(\omega_{\mathrm{T}}-\omega) \\ &+ \left(\frac{\Delta\omega}{2} - \kappa_0\Omega\right)\left[\left(1 \mp \frac{1}{\sqrt{2}}\right)\gamma^2 + \kappa_0^2\Omega^2 + \frac{\Delta\omega^2}{4}\right] = 0. \end{aligned} \tag{S.73}$$

Solving the cubic equation in terms of $(\omega_{\mathrm{T}}-\omega)$ yields the Ω to ω_{T} transduction function at the cusp singularities $X_{1,2}$, expressed as

$$\omega_{\mathrm{T}}(\Omega) = \omega - \frac{1}{3}(\kappa_0\Omega - \frac{\Delta\omega}{2}) + \sqrt[3]{-\frac{P_0'}{2} + \sqrt{\frac{P_0'^2}{4} + \frac{P_1'^3}{27}}} + \sqrt[3]{-\frac{P_0'}{2} - \sqrt{\frac{P_0'^2}{4} + \frac{P_1'^3}{27}}}, \tag{S.74}$$

where

$$\begin{aligned} P_1' &= -\frac{1}{3}\left(4\kappa_0^2\Omega^2 + \Delta\omega^2 - \Delta\omega\kappa_0\Omega\right), \\ P_0' &= \left(\frac{\Delta\omega}{2} - \kappa_0\Omega\right)\left[\frac{2}{27}(64\kappa_0^2\Omega^2 + 2\Delta\omega^2 + \Delta\omega\kappa_0\Omega) + \left(1 \mp \frac{1}{\sqrt{2}}\right)\gamma^2\right], \end{aligned} \tag{S.75}$$

The sign '$-$' ('+') in '$\mp$' is associated with X_1 (X_2).

When the condition of degeneracy $\Delta\omega = 0$ is met, the governing equation for ω_{T} in equation (S.73) simplifies further to

$$(\omega_{\mathrm{T}}-\omega)^3 + \kappa_0\Omega(\omega_{\mathrm{T}}-\omega)^2 - \kappa_0^2\Omega^2(\omega_{\mathrm{T}}-\omega) - \kappa_0\Omega\left[\left(1 \mp \frac{1}{\sqrt{2}}\right)\gamma^2 + \kappa_0^2\Omega^2\right] = 0. \tag{S.76}$$

Solving it allows us to express the Ω to ω_{T} transduction function more directly,

$$\begin{aligned}\omega_{\mathrm{T}}(\Omega) = \omega &- \frac{1}{3}\kappa_0\Omega \\ &+ \left\{\frac{8}{27}\kappa_0^3\Omega^3 + \frac{1}{2}\kappa_0\Omega\left(1 \mp \frac{1}{\sqrt{2}}\right)\gamma^2 + \kappa_0\Omega\gamma\left[\frac{1}{4}\left(1 \mp \frac{1}{\sqrt{2}}\right)^2\gamma^2 + \frac{8}{27}\kappa_0^2\Omega^2\left(1 \mp \frac{1}{\sqrt{2}}\right)\right]^{1/2}\right\}^{1/3} \\ &+ \left\{\frac{8}{27}\kappa_0^3\Omega^3 + \frac{1}{2}\kappa_0\Omega\left(1 \mp \frac{1}{\sqrt{2}}\right)\gamma^2 - \kappa_0\Omega\gamma\left[\frac{1}{4}\left(1 \mp \frac{1}{\sqrt{2}}\right)^2\gamma^2 + \frac{8}{27}\kappa_0^2\Omega^2\left(1 \mp \frac{1}{\sqrt{2}}\right)\right]^{1/2}\right\}^{1/3}. \end{aligned} \tag{S.77}$$

Also, the sign '$-$' ('+') in '$\mp$' is associated with sensing at X_1 (X_2).

In Fig. 3a,b of the main text, the theoretical frequency outputs at $X_{1,2}$ (depicted by red and blue curves) are derived using the transduction function (S.77). The logarithmic plots with 1/3 slopes verify that Ω perturbations near $X_{1,2}$ yield frequency outputs on the order of $\Omega^{1/3}$.

The fluctuations in the natural frequency ω can directly affect the rotation-induced phase-tracked frequency $\omega_{\mathrm{T}}(\Omega)$, a primary source of error in frequency-output operations. Additionally, static voltages govern the system's coupling g and degeneracy $\Delta\omega$ via the electrostatic effect, where fluctuations can lead to deviations in frequency outputs. External factors like temperature or stress may further introduce parametric errors to the gyroscope.

We also investigate how *coupling perturbations influence the phase-tracked frequency ω_{T} near cusp singularities*. By setting $\Omega = 0$ and $\Delta\omega = 0$ in the governing equation for ω_{T} (S.42), the relation between g and ω_{T} is derived as:

$$\left[(\omega_{\mathrm{T}} - \omega)^2 + \frac{\gamma^2}{4} - \frac{g\gamma}{2} - \frac{g^2}{4}\right](\omega_{\mathrm{T}} - \omega) = 0, \tag{S.78}$$

resulting in a "pitchfork" bifurcation depicted in Fig. 1c or d.

For $(-\sqrt{2} - 1)\gamma < g < (\sqrt{2} - 1)\gamma$, equation (S.78) yields a sole solution of $\omega_{\mathrm{T}} = \omega$ with no g dependency. Conversely, if g is either $\leq (-\sqrt{2} - 1)\gamma$ or $\geq (\sqrt{2} - 1)\gamma$, equation (S.78) offers one unstable solution $\omega_{\mathrm{T}} = \omega$ and two stable ones $\omega_{\mathrm{T}} = \omega \pm \frac{1}{2}\sqrt{g^2 + 2g\gamma - \gamma^2}$, which are approximately proportional to $g^{1/2}$ near $X_{1,2}$.

SUPPLEMENTARY NOTE 9. CUSP-SINGULARITY-MEDIATED PHASE MODULATION

In this section, we delve into another compelling variable of the phase-tracked system, the *relative phase ϑ*, defined by equation (S.64). Figure 4a in the main text theoretically depicts ϑ as a function of the coupling strength g and angular velocity Ω. This depiction combines equation (S.64) with the phase-tracked frequency's governing equation ω_{T} (S.42), while removing the intermediary variable $(\omega_{\mathrm{T}} - \omega)$:

$$\begin{cases} \vartheta = \mathrm{Arg}\left\{g(\omega_{\mathrm{T}} - \omega + \kappa_0\Omega) - \frac{\Delta\omega}{2}\gamma + \mathrm{i}\left[(\omega_{\mathrm{T}} - \omega + \kappa_0\Omega)^2 - \frac{\Delta\omega^2}{4} + \frac{\gamma^2}{4} - \frac{g^2}{4}\right]\right\}, \\ -(\omega_{\mathrm{T}} - \omega)^3 - \left(\kappa_0\Omega - \frac{\Delta\omega}{2}\right)(\omega_{\mathrm{T}} - \omega)^2 - \left(\frac{\gamma^2}{4} - \frac{g\gamma}{2} - \frac{g^2}{4} - \kappa_0^2\Omega^2 - \frac{\Delta\omega^2}{4}\right)(\omega_{\mathrm{T}} - \omega) \\ -\left(\frac{\Delta\omega}{2} - \kappa_0\Omega\right)\left(\frac{\gamma^2}{4} + \frac{g^2}{4} + \kappa_0^2\Omega^2 + \frac{\Delta\omega^2}{4}\right) = 0. \end{cases}$$

The *ϑ surface exhibits cusp singularities at $X_{1,2}$, which promise high sensitivity in transduction*. This study focuses on ϑ's response to angular rotation Ω.

We now examine the degenerate scenario $\Delta\omega = 0$. In standard QFM operation, or $g = 0$, the relative phase remains unchanged and is unaffected by Ω, specifically $\vartheta = \mathrm{Arg}(\mathrm{i}) = \pi/2$. Conversely, for $g =$

$(\pm\sqrt{2}-1)\gamma$, the relative phase ϑ is given by

$$\vartheta = \mathrm{Arg}\left\{(\pm\sqrt{2}-1)\gamma(\omega_{\mathrm{T}}-\omega+\kappa_0\Omega)+\mathrm{i}\left[(\omega_{\mathrm{T}}-\omega+\kappa_0\Omega)^2+\frac{\pm\sqrt{2}-1}{2}\gamma^2\right]\right\}, \tag{S.79}$$

where ω_{T} is provided in (S.76):

$$(\omega_{\mathrm{T}}-\omega)^3+\kappa_0\Omega(\omega_{\mathrm{T}}-\omega)^2-\kappa_0^2\Omega^2(\omega_{\mathrm{T}}-\omega)-\kappa_0\Omega\left[\left(1\mp\frac{1}{\sqrt{2}}\right)\gamma^2+\kappa_0^2\Omega^2\right]=0.$$

The relative phase ϑ at the *critical coupling strengths* $g=(\pm\sqrt{2}-1)\gamma$ as a function of Ω can be obtained by substituting the associated singularity-mediated frequency $\omega_{\mathrm{T}}(\Omega)$ (S.77) into (S.79). The red and blue curves in Fig. 4b,c of the main text illustrate that ϑ is highly sensitive at $g=(\pm\sqrt{2}-1)\gamma$. The logarithmic plots display a slope of 1/3, signifying that these *phase outputs exhibit cubic-root responsivities*, such that $\vartheta \propto \Omega^{1/3}$.

In practical terms, the relative phase ϑ is deduced by the subtraction of phase signals of modes 2 and 1, specifically $\vartheta=\theta_2-\theta_1$. Given that θ_1 is precisely maintained at a constant $-\pi/2$ during the measurements, θ_2, which we measure directly, can also serve as the effective phase output.

The theoretical model of ϑ remains uncorrelated with the system's natural frequencies, meaning that natural-frequency fluctuation, the primary source of error in frequency outputs, does not impact the phase output. Consequently, *phase outputs exhibit higher precision than frequency outputs*, as evidenced by bias measurements. Nonetheless, phase outputs are susceptible to instability due to the fluctuations in coupling and degeneracy tuning voltages or parametric errors from external disturbances, which we consider principal causes of such instability.

As Ω varies from negative to positive infinity, the evolution of the phase-tracked states at particular coupling strengths $g=(\sqrt{2}-1)\gamma$, $-\gamma$, and $(-\sqrt{2}-1)\gamma$ are depicted as blue, purple, and red paths on the Poincaré sphere in Supplementary Fig. **6**a, respectively. These trajectories, when projected onto the S_1-S_2 equatorial plane of the Poincaré sphere, form three closed loops (Supplementary Fig. **6**b). The topology of these loops is characterized by the *winding number*:

$$\nu=\frac{1}{2\pi}\int_{-\infty}^{\infty}\vartheta\,\mathrm{d}\Omega, \tag{S.80}$$

which quantifies the *number of encirclements of the origin* in the S_1-S_2 plane. The red (blue) loop pertains to the Ω measurements at X_2 (X_1), resulting in a -2π (0) accumulation of ϑ, indicating a winding number of $\nu=-1$ ($\nu=0$). The purple loop intersects the coordinate $(0,0,1)$, representing a ϑ-phase singularity PS_2 with a topological charge of 1 located at $[g=-\gamma,\Omega=0]$. Adjusting g to pass through the phase singularity PS_2 induces a topological phase transition, denoted by a shift in ν. The cusp singularity at X_2 (X_1) is situated within the nontrivial (trivial) topological phase.

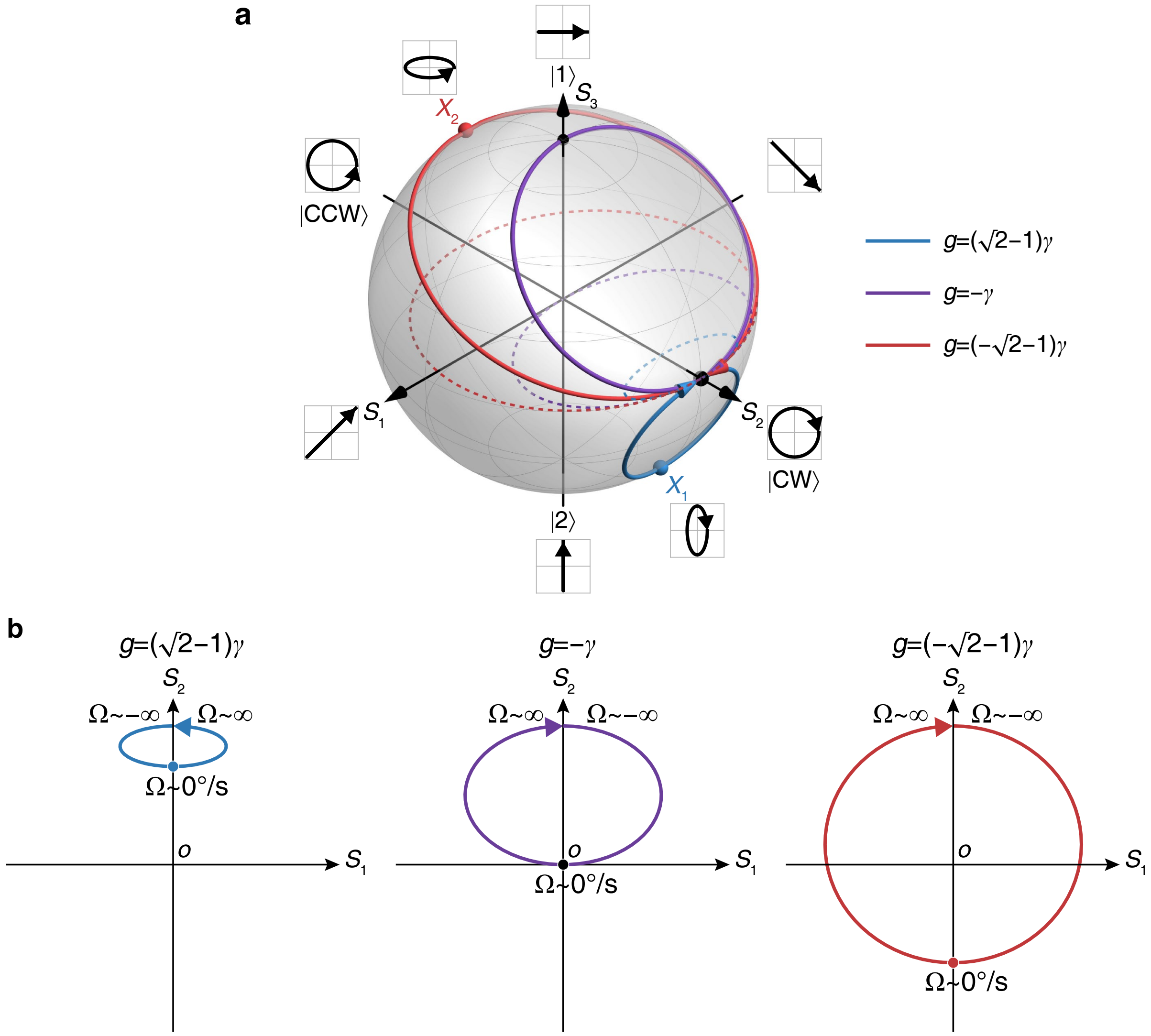

Supplementary Figure 6. **Topologies of the phase-tracked state evolutions when the angular velocity Ω changes from negative to positive infinity at constant couplings $g = (\sqrt{2} - 1)\gamma$, $-\gamma$ or $(-\sqrt{2} - 1)\gamma$. a**, State evolutions on the Poincaré sphere. **b**, Trajectory projections from (**a**) onto the S_1-S_2 equatorial plane, forming three closed loops. The arrows indicate the increasing-Ω direction.

SUPPLEMENTARY NOTE 10. ANGULAR-VELOCITY ESTIMATION

Estimating the angular velocity from the measured frequency or phase outputs is crucial, particularly since this task is more complex than standard linear sensing, due to the nonlinearity of singularity-mediated transduction functions. We present theoretical models for converting frequency or phase outputs to angular velocity inputs, assuming $\Delta\omega = 0$ throughout the subsequent analyses.

Initially, we examine the frequency output process facilitated by singularities, dictated by equation (S.76). In this context, the angular velocity Ω functions as the dependent variable, with (S.76) serving as a cubic equation about $\kappa_0\Omega$:

$$\kappa_0^3\Omega^3 + (\omega_\mathrm{T} - \omega)\kappa_0^2\Omega^2 + \left[\left(1 \mp \frac{1}{\sqrt{2}}\right)\gamma^2 - (\omega_\mathrm{T} - \omega)^2\right]\kappa_0\Omega - (\omega_\mathrm{T} - \omega)^3 = 0. \tag{S.81}$$

Solving this equation yields the ω_T to Ω estimation function,

$$\begin{aligned}\kappa_0\Omega = &-\frac{1}{3}\delta \\ &+ \left\{\frac{8}{27}\delta^3 + \frac{1}{6}\left(1 \mp \frac{1}{\sqrt{2}}\right)\gamma^2\delta + \left[\frac{1}{27}\left(1 \mp \frac{1}{\sqrt{2}}\right)^3\gamma^6 - \frac{13}{108}\left(1 \mp \frac{1}{\sqrt{2}}\right)^2\gamma^4\delta^2 + \frac{24}{81}\left(1 \mp \frac{1}{\sqrt{2}}\right)\gamma^2\delta^4\right]^{1/2}\right\}^{1/3} \\ &+ \left\{\frac{8}{27}\delta^3 + \frac{1}{6}\left(1 \mp \frac{1}{\sqrt{2}}\right)\gamma^2\delta - \left[\frac{1}{27}\left(1 \mp \frac{1}{\sqrt{2}}\right)^3\gamma^6 - \frac{13}{108}\left(1 \mp \frac{1}{\sqrt{2}}\right)^2\gamma^4\delta^2 + \frac{24}{81}\left(1 \mp \frac{1}{\sqrt{2}}\right)\gamma^2\delta^4\right]^{1/2}\right\}^{1/3},\end{aligned} \tag{S.82}$$

where $\delta \equiv \omega_\mathrm{T} - \omega$. The sign '$-$' ('+') in '$\mp$' is associated with sensing at X_1 (X_2).

Subsequently, we consider the singularity-mediated phase output operation, dictated by these equations,

$$\begin{cases} \vartheta = \mathrm{Arg}\left\{(\pm\sqrt{2} - 1)\gamma(\delta + \kappa_0\Omega) + \mathrm{i}\left[(\delta + \kappa_0\Omega)^2 + \frac{\pm\sqrt{2}-1}{2}\gamma^2\right]\right\}, \\ \delta^3 + \kappa_0\Omega\delta^2 - \kappa_0^2\Omega^2\delta - \kappa_0\Omega[(1 \mp \frac{1}{\sqrt{2}})\gamma^2 + \kappa_0^2\Omega^2] = 0. \end{cases}$$

By defining a new intermediate variable $\zeta \equiv \omega_\mathrm{T} - \omega + \kappa_0\Omega$, the ϑ to Ω estimation function is given by

$$\begin{cases} \kappa_0\Omega = \frac{\zeta^3}{2\zeta^2 - (\pm\frac{1}{\sqrt{2}} - 1)\gamma^2}, \\ \tan\vartheta = \frac{\zeta^2 + \frac{1}{2}(\pm\sqrt{2} - 1)\gamma^2}{(\pm\sqrt{2} - 1)\gamma\zeta}, \end{cases} \tag{S.83}$$

with '+' ('$-$') in '$\pm$' associated with sensing at X_1 (X_2).

SUPPLEMENTARY NOTE 11. BROWNIAN NOISE MODEL FOR SINGULARITY-ENHANCED FREQUENCY AND PHASE MODULATIONS

The Brownian noise, or thermomechanical noise, of the resonator sets the fundamental performance limit of a gyroscope. Here, we analyse how the *Brownian motion affects the singularity-enhanced frequency and phase modulations*. Brownian noise arises from an effective Gaussian white fluctuating force exerted by the thermal reservoir on the operational modes. The fluctuation-dissipation theorem gives the spectral density of this force as [15]

$$S_{\text{fn}} = 2k_{\text{B}}Tm\gamma, \tag{S.84}$$

where k_{B} is the Boltzmann constant, T is the ambient temperature, m is the modal mass, and γ is the dissipation rate.

Within the framework of the Heisenberg-Langevin equation (S.34), the spectral density of the reduced thermal Langevin force ξ_j acting on mode $j = 1, 2$ is [15]

$$S_{\xi_j} = \frac{S_{\text{fn}}}{2\hbar m\omega_j} = \frac{k_{\text{B}}T\gamma}{\omega_j}, \tag{S.85}$$

under the assumption of $\hbar = 1$.

Including the Langevin forces, the complete Heisenberg-Langevin equations of motion for the gyroscope become

$$\begin{bmatrix} \dot{a}_1 \\ \dot{a}_2 \end{bmatrix} = \begin{bmatrix} \mathrm{i}\omega_1 - \gamma/2 & \mathrm{i}g/2 + \kappa_0\Omega \\ \mathrm{i}g/2 - \kappa_0\Omega & \mathrm{i}\omega_2 - \gamma/2 \end{bmatrix} \begin{bmatrix} a_1 \\ a_2 \end{bmatrix} - \begin{bmatrix} \mathrm{i} \\ -1 \end{bmatrix} f\mathrm{e}^{\mathrm{i}\omega_{\mathrm{d}}t} + \begin{bmatrix} \xi_1 \\ \xi_2 \end{bmatrix}. \tag{S.86}$$

To analyse the frequency and phase noise caused by the Langevin forces $\xi_{1,2}$, we employ phasor analysis [16] by writing the complex amplitudes $a_{1,2}$ as

$$a_j(t) = |a_j(t)|\mathrm{e}^{\mathrm{i}\Theta_j(t)}, \tag{S.87}$$

where the amplitudes and phases are time-dependent real variables, $|a_j(t)|, \Theta_j(t) \in \mathbb{R}$. The derivatives of a_j with respect to time are given by

$$\dot{a}_j = (|\dot{a}_j| + \mathrm{i}|a_j|\dot{\Theta}_j)\mathrm{e}^{\mathrm{i}\Theta_j}. \tag{S.88}$$

We can further expand the time-dependent phases as $\Theta_j(t) = \omega_{\mathrm{d}}t + \theta_j$, which gives $\Theta_2 - \Theta_1 = \vartheta$ and $\dot{\Theta}_j = \dot{\theta}_j$.

Substituting (S.87) and (S.88) into (S.86) gives the differential equations for $|a_j|$ and θ_j:

$$|\dot{a}_1| = -\gamma/2|a_1| + (\kappa_0\Omega\cos\vartheta - g/2\sin\vartheta)|a_2| - f\sin\theta_1 + \xi_{1,\mathrm{c}}, \tag{S.89}$$

$$|\dot{a}_2| = -\gamma/2|a_2| - (\kappa_0\Omega\cos\vartheta - g/2\sin\vartheta)|a_1| + f\cos\theta_2 + \xi_{2,\mathrm{c}}, \tag{S.90}$$

$$\dot{\theta}_1 = \omega_1 - \omega_\mathrm{d} + (\kappa_0\Omega\sin\vartheta + g/2\cos\vartheta)|a_2|/|a_1| - f/|a_1|\cos\theta_1 + \xi_{1,\mathrm{s}}/|a_1|, \tag{S.91}$$

$$\dot{\theta}_2 = \omega_2 - \omega_\mathrm{d} + (\kappa_0\Omega\sin\vartheta + g/2\cos\vartheta)|a_1|/|a_2| - f/|a_2|\sin\theta_2 + \xi_{2,\mathrm{s}}/|a_2|. \tag{S.92}$$

The amplitude- and phase-quadrature noise spectral densities of the Langevin forces $\xi_{\mathrm{j,c}}$, $\xi_{\mathrm{j,s}}$ satisfy

$$S_{\xi_{j,\mathrm{c}}} = S_{\xi_{j,\mathrm{s}}} = \frac{S_{\xi_j}}{2} = \frac{k_\mathrm{B}T\gamma}{2\omega_j}, \tag{S.93}$$

We impose the $\theta_1 = -\pi/2$ PhT condition and degeneracy ($\omega_1 = \omega_2 = \omega$) to (S.89-S.92). When the PhT system is operated at cusp singularities $X_{1,2}$ without rotation, we have $g = (\pm\sqrt{2} - 1)\gamma$, $\vartheta = \pm\pi/2$, and $\Omega = 0$. Equations (S.91) and (S.92) then reduce to

$$\dot{\theta}_1 = \omega - \omega_\mathrm{T} + \xi_{1,\mathrm{s}}/|a_1|, \tag{S.94}$$

$$\dot{\theta}_2 = \omega - \omega_\mathrm{T} + \xi_{2,\mathrm{s}}/|a_2|. \tag{S.95}$$

We focus on the frequency noise from $\xi_{\mathrm{j,s}}$, and neglect the small amplitude-noise contributions entering via $\xi_{\mathrm{j,s}}/|a_j|$. Based on the relationship between a_j and q_j described by (S.36), we have

$$|a_j| = \sqrt{\frac{m\omega_j}{2}}|q_j|. \tag{S.96}$$

a. Frequency-modulated readout. First, we analyse the Brownian noise in the singularity-enhanced frequency modulation. The PhT oscillation frequency ω_T is governed by (S.94). The Brownian frequency noise $\xi_{1,\mathrm{s}}/|a_1|$ adds directly to ω_T, with spectral density

$$S_{\omega_{\mathrm{T,n}}} = \frac{k_\mathrm{B}T}{m\omega Q|q_1|^2}, \tag{S.97}$$

where $Q = \omega/\gamma$ is the quality factor of the degenerate operational modes. It is noteworthy that the spectral density of the Brownian frequency noise (S.97) in this singularity-enhanced case is identical to that of the conventional frequency-modulated operations [16].

b. Phase-modulated readout. Next, we analyse the Brownian noise in the singularity-mediated phase modulation. The phase noise $\theta_{j,\mathrm{n}}$ is the time integral of the frequency noise $\dot{\theta}_{j,\mathrm{n}}$, that is $\theta_{j,\mathrm{n}} = \int \dot{\theta}_{j,\mathrm{n}}\mathrm{d}t$. In the frequency domain, we have $\theta_{j,\mathrm{n}}(\tilde{\omega}) = \frac{\dot{\theta}_{j,\mathrm{n}}(\tilde{\omega})}{\mathrm{i}\tilde{\omega}}$, where $\tilde{\omega}$ denotes the general angular frequency. Therefore, the phase-noise spectral density is suppressed by $1/\tilde{\omega}^2$ relative to the frequency-noise spectral density. Near resonance, $\tilde{\omega} \approx \omega$, the spectral density of the Brownian phase noise in θ_j is given by

$$S_{\theta_{j,\mathrm{n}}} = \frac{S_{\dot{\theta}_{j,\mathrm{n}}}}{\omega^2} = \frac{k_\mathrm{B}T}{m\omega^3 Q|q_j|^2}. \tag{S.98}$$

The measurement of ϑ involves the detection of both θ_1 and θ_2, so the spectral density of the Brownian phase noise in ϑ is the sum of $S_{\theta_{1,\text{n}}}$ and $S_{\theta_{2,\text{n}}}$, $S_{\vartheta_\text{n}} = S_{\theta_{1,\text{n}}} + S_{\theta_{2,\text{n}}}$. At X_1, $|q_2|/|q_1| = \sqrt{2}+1$, and the spectral density of the Brownian phase noise of ϑ is expressed as

$$S_{\vartheta_\text{n}}^{X_1} = \sqrt{2}\frac{k_\text{B}T}{m\omega^3 Q|q_1|^2}. \tag{S.99}$$

At X_2, $|q_2|/|q_1| = \sqrt{2} - 1$, and the spectral density of the Brownian phase noise of ϑ is otherwise given by

$$S_{\vartheta_\text{n}}^{X_2} = (\sqrt{2}+2)\frac{k_\text{B}T}{m\omega^3 Q|q_1|^2}. \tag{S.100}$$

In summary, Eqs. (S.91) and (S.92) show that Brownian frequency and phase noises enter additively in the corresponding output channels and are **not** amplified by the singularities. By contrast, the Coriolis-induced frequency and phase modulations are enhanced at the cusp singularities. Consequently, the sensing signal is boosted while the *noise floor remains nearly unchanged*, yielding an improved signal-to-noise ratio.

SUPPLEMENTARY NOTE 12. PERFORMANCE LIMIT OF AMPLITUDE-MODULATED OPERATION

Additionally, it is essential to compare with the conventional amplitude-modulation (AM), the predominant commercial mode for CVGs.

First, we introduce the function of the AM gyroscope. In AM operation, mode 1 is driven to oscillate at a steady amplitude, typically using a phase-locked loop and an amplitude controller. We now aim to deduce the angular velocity from the mode-2 displacement amplitude, induced by the Coriolis force from mode 1. However, mode 2 also feeds energy back to mode 1 via the Coriolis effect, complicating the system. This coherent coupling, represented by the off-diagonal Hamiltonian elements (S.23), leads to normal mode splitting. Beyond the weak-strong critical coupling strength (which is $2\kappa_0\Omega = \gamma$ for degeneracy or higher, if not), mode-2 amplitude experiences saturation, limiting the Ω measurement range. To address this, an extra controlling force suppresses the mode-2 motion, negating the Coriolis feedback to mode 1 and creating a non-reciprocal coupling configuration. In this so-called *force-to-rebalance* AM mode, angular velocity is determined by tracking the mode-2 suppressing force strength.

The precision of the AM gyroscope highly relies on the quality of displacement transduction and control. In the following, we employ recognized theoretical models [17, 18] to estimate the theoretical limits of the angle random walk (ARW) and bias stability of the disk resonator in this study when configured as an AM gyroscope.

A. Angle-random-walk limit of amplitude-modulated operation

The noise characteristics of an AM-mode CVG arise from two main sources: Brownian (thermomechanical) noise and electronic noise, as given by $\text{ARW} = \sqrt{\text{ARW}^2_{\text{Brownian}} + \text{ARW}^2_{\text{Electronic}}}$. Electronic noise, $\text{ARW}_{\text{Electronic}}$, is related to displacement transduction efficiency and the quality of measurement and control circuits. Brownian noise, $\text{ARW}_{\text{Brownian}}$, is due to the thermomechanical interaction with the environment, representing the fundamental limit of the device.

The Brownian noise $\text{ARW}_{\text{Brownian}}$ aids in assessing the theoretical boundary for the AM-mode noise performance of the resonator, as well-defined in Ref. [18] and presented by

$$\text{ARW}_{\text{Brownian}} = \frac{1}{2\kappa_0 |q_{\text{drive}}|}\sqrt{\frac{4k_{\text{B}}T}{\omega_0 m Q}} \times \frac{180}{\pi} \times 60 \ (^\circ/\sqrt{\text{h}}). \tag{S.101}$$

Here, $|q_{\text{drive}}|$, the displacement amplitude of the driven mode, is approximately 0.5 μm for our resonator. The resonant frequency, effective proof mass, and quality factor for the disk resonator's degenerate modes are $\omega_0 \approx 2\pi \times 40.4$ kHz, $m \approx 60$ μg, and $Q \approx 111.8$ k, respectively.

In AM mode, the disk resonator in this investigation is calculated to yield a Brownian noise performance limit of approximately $\text{ARW}_{\text{Brownian}} \approx 0.018^\circ/\sqrt{\text{h}}$. Including electronic noise results in an increased overall angle random walk, $\text{ARW} > 0.018^\circ/\sqrt{\text{h}}$.

B. Bias-stability limit of AM operation

The theoretical limit of the bias stability $\delta\Omega$ in the standard mode-matched AM silicon CVG is effectively approximated by [17]

$$\delta\Omega = \text{FOM} \times \delta P, \tag{S.102}$$

where the figure of merit (FOM) is given by

$$\text{FOM} = \frac{1}{\kappa_0 \tau_0} \times \frac{180}{\pi} \ (^\circ/\text{s}), \tag{S.103}$$

and $\tau_0 = 2/\gamma$ represents the decay time constant of the modes. The parameter δP accounts for effective structural variations due to environmental factors, such as temperature or stress, with a widely accepted value of $\delta P \approx 0.8$ ppm, used consistently to project or assess bias stability in standard AM mode silicon on-chip CVGs [17].

In AM mode operation, the disk resonator in this study, with an intrinsic Coriolis factor $\kappa_0 = 0.588$ and a dissipation rate $\gamma = 2\pi \times 0.36$ Hz, is expected to achieve a bias stability of $0.32^\circ/\text{h}$. This typically

represents the highest precision the resonator can attain. Practical errors in displacement transduction and electronic control add further drift, resulting in a worse overall bias drift, $\delta\Omega > 0.32°/\mathrm{h}$.

In summary, *the performance of the singularity-mediated phase output can significantly exceed traditional AM operation by almost two orders of magnitude in ARW and one order of magnitude in bias stability.*

SUPPLEMENTARY REFERENCES.

[1] R. Antonello and R. Oboe, MEMS gyroscopes for consumers and industrial applications, in *Microsensors*, edited by I. Minin (IntechOpen, Rijeka, 2011) Chap. 12.

[2] C. Acar and A. Shkel, *MEMS Vibratory Gyroscopes: Structural Approaches to Improve Robustness*, MEMS Reference Shelf (Springer New York, NY, 2009).

[3] J. Campanile, Enhanced scaleable SIRU, in *2016 IEEE/ION Position, Location and Navigation Symposium (PLANS)*, Vol. 86 (2016) pp. 905–909.

[4] M. W. Putty, *A micromachined vibrating ring gyroscope*, Ph.D. thesis, University of Michigan, Ann Arbor, MI (1995).

[5] J. Y. Cho, *High-performance micromachined vibratory rate- and rate-integrating gyroscopes*, Ph.D. thesis, University of Michigan, Ann Arbor, MI (2012).

[6] K. Li, H. Fu, and Y. Li, Coriolis-force-induced coupling between two modes of a mechanical resonator for detection of angular velocity, Physical Review A **98**, 023862 (2018).

[7] O. V. Ivakhnenko, S. N. Shevchenko, and F. Nori, Simulating quantum dynamical phenomena using classical oscillators: Landau-Zener-Stückelberg-Majorana interferometry, latching modulation, and motional averaging, Scientific Reports **8**, 12218 (2018).

[8] H. Xu, D. Mason, L. Jiang, and J. G. E. Harris, Topological energy transfer in an optomechanical system with exceptional points, Nature **537**, 80 (2016).

[9] H. Okamoto, A. Gourgout, C.-Y. Chang, K. Onomitsu, I. Mahboob, E. Y. Chang, and H. Yamaguchi, Coherent phonon manipulation in coupled mechanical resonators, Nature Physics **9**, 598 (2013).

[10] S. Assawaworrarit, X. Yu, and S. Fan, Robust wireless power transfer using a nonlinear parity–time-symmetric circuit, Nature **546**, 387 (2017).

[11] R. Thom, *Structural stability and morphogenesis* (Benjamin, 1975).

[12] V. I. Arnold, *Catastrophe Theory* (Springer-Verlag, 1984).

[13] R. Fleury, D. L. Sounas, C. F. Sieck, M. R. Haberman, and A. Alù, Sound isolation and giant linear nonreciprocity in a compact acoustic circulator, Science **343**, 516 (2014).

[14] D. Pan, H. Xu, and F. J. G. de Abajo, Rotational Doppler cooling and heating, Science Advances **7**, eabd6705 (2021).

[15] E. Thuneberg, *Quantum optics in electric circuits* (University of Oulu, 2017).

[16] M. Kline, *Frequency modulated gyroscopes*, Ph.D. thesis, University of California at Berkeley, Berkeley, CA (2015).

[17] A. D. Challoner, H. G. Howard, and J. Y. Liu, Boeing disc resonator gyroscope, in *IEEE/ION Position, Location and Navigation Symposium-PLANS 2014* (IEEE/ION, Monterey, CA, USA, 2014) pp. 504–514.

[18] F. Ayazi and K. Najafi, A HARPSS polysilicon vibrating ring gyroscope, Journal of Microelectromechanical Systems **10**, 169 (2001).
